# MDM4 inhibition: a novel therapeutic strategy to reactivate p53 in hepatoblastoma

**DOI:** 10.1038/s41598-021-82542-4

**Published:** 2021-02-03

**Authors:** Sarah E. Woodfield, Yan Shi, Roma H. Patel, Zhenghu Chen, Aayushi P. Shah, Rohit K. Srivastava, Richard S. Whitlock, Aryana M. Ibarra, Samuel R. Larson, Stephen F. Sarabia, Andrew Badachhape, Zbigniew Starosolski, Ketan B. Ghaghada, Pavel Sumazin, D. Allen Annis, Dolores López-Terrada, Sanjeev A. Vasudevan

**Affiliations:** 1 Divisions of Pediatric Surgery and Surgical Research, Michael E. DeBakey Department of Surgery, Pediatric Surgical Oncology Laboratory, Texas Children’s Surgical Oncology Program, Texas Children’s Liver Tumor Program, Dan L. Duncan Cancer Center, Baylor College of Medicine, Texas Children’s Hospital, 1102 Bates Ave., Suite 460G, Houston, TX 77030-2399 USA; 2 Department of Pathology and Immunology, Baylor College of Medicine, Molecular Oncology Laboratory, Texas Children’s Hospital, Houston, TX 77030 USA; 3 Singleton Department of Pediatric Radiology, Texas Children’s Hospital, Houston, TX 77030 USA; 4 Department of Pediatrics, Dan L. Duncan Cancer Center, Baylor College of Medicine, Houston, TX 77030 USA; 5 Aileron Therapeutics Inc, Cambridge, MA 02139 USA

**Keywords:** Targeted therapies, Liver cancer

## Abstract

Hepatoblastoma (HB) is the most common pediatric liver malignancy. High-risk patients have poor survival, and current chemotherapies are associated with significant toxicities. Targeted therapies are needed to improve outcomes and patient quality of life. Most HB cases are *TP53* wild-type; therefore, we hypothesized that targeting the p53 regulator Murine double minute 4 (MDM4) to reactivate p53 signaling may show efficacy. *MDM4* expression was elevated in HB patient samples, and increased expression was strongly correlated with decreased expression of p53 target genes. Treatment with NSC207895 (XI-006), which inhibits MDM4 expression, or ATSP-7041, a stapled peptide dual inhibitor of MDM2 and MDM4, showed significant cytotoxic and antiproliferative effects in HB cells. Similar phenotypes were seen with short hairpin RNA (shRNA)-mediated inhibition of *MDM4*. Both NSC207895 and ATSP-7041 caused significant upregulation of p53 targets in HB cells. Knocking-down *TP53* with shRNA or overexpressing *MDM4* led to resistance to NSC207895-mediated cytotoxicity, suggesting that this phenotype is dependent on the MDM4-p53 axis. MDM4 inhibition also showed efficacy in a murine model of HB with significantly decreased tumor weight and increased apoptosis observed in the treatment group. This study demonstrates that inhibition of MDM4 is efficacious in HB by upregulating p53 tumor suppressor signaling.

## Introduction

Liver cancer remains one of the leading causes of cancer-related deaths worldwide^1^. Pediatric liver malignancies affect the lives of many children with hepatoblastoma (HB) being the most common, particularly in the setting of prematurity and very low birth weight infants^2–4^. More recent data has shown that the worldwide incidence of HB is increasing at a rate higher than any other pediatric malignancy^5^; moreover, there is a much higher incidence of HB in children born with non-chromosomal congenital defects^6^. HB typically afflicts patients less than four years of age and has an overall survival (OS) rate approaching 80% at five years after diagnosis^7^; however, patients with high-risk, metastatic disease have a five-year OS rate of only about 40%^7^. The best chance of survival and mainstay of oncologic therapy for patients with progressive and metastatic HB is complete surgical resection with negative margins^8^. Current high dose, non-targeted chemotherapy for HB, including mainly doxorubicin and cisplatin, is effective in increasing resectability; however, complications of treatment-related deafness, renal toxicity, cardiotoxicity, and neutropenia contribute to morbidity and mortality in this setting^9–11^. At present there are no approved targeted therapies for HB patients. Thus, a primary research goal is to design new, less toxic therapies targeting vulnerable pathways to make more tumors amenable to surgical resection and to increase the cure rate of children with HB.

In response to intra- and extracellular stresses, such as genotoxic stress and alterations in DNA, the p53 pathway is activated to induce cell cycle arrest, cellular senescence, and apoptosis in order to cease the propagation of mutations^12^. Specifically, the p53 protein is known to function upstream of apoptosis through transcriptional activation of *Bax* and *Puma* and of cell cycle arrest and senescence through *Cyclin dependent kinase inhibitor 1A* (*CDKN1A*)^13^. The *TP53* gene has been identified as the most frequently inactivated gene in cancer and is mutated in approximately 50% of all cancers^12^. Interestingly, hepatocellular malignancies have a paucity of *TP53* mutations. Mutations in the *TP53* gene were seen in only 31% of adult hepatocellular carcinoma (HCC) cases in a recent study profiling 363 HCC cases^14^. In comparison, HB cases have shown almost no mutations in this gene in single strand conformation polymorphism analysis and exome sequencing studies^15,16^. Notably, HB tumors have a very low mutation rate of about 2.9 mutations per tumor with recurrent mutations most commonly seen in the *CTNNB1* gene that codes for the β-catenin protein^15^. Additionally, normal hepatocytes are not susceptible to p53-mediated apoptosis^17^. Therefore, targeting the major regulators of p53 function to reactivate the pathway is an attractive therapeutic strategy for *TP53* wild-type HB.

The major regulators of p53 expression and function are Murine double minute 2 (MDM2) and MDM4 (MDMX). MDM2 and MDM4 work together and independently to inhibit and degrade the p53 tumor suppressor protein. MDM2 is known to monoubiquitinate the p53 protein; however, polyubiquitination of p53 requires heterodimer formation between MDM2 and MDM4^18^. In addition, MDM4 can directly inhibit p53 transcriptional activity by binding the transactivation domain and by directly binding and degrading p21^12^. *MDM4* amplification and overexpression have been shown in many cancer types, such as melanoma, breast cancer, glioma, and soft tissue sarcoma^12^. Copy number gain of the 1q32.1 chromosomal region was shown in cases of HB and HCC, including in 28 of 56 HB tumors in a study that showed that this was the most frequent allelic imbalance in HB tumors, and *MDM4* has been proposed as the candidate oncogene in this amplicon^19–21^. Biomarker studies using immunoblotting have shown that expression of MDM4 in HCC correlates with a poor prognosis^22^. In addition, a study of 363 HCC cases showed that *MDM4* expression and copy number were significantly increased in cases with wild-type *TP53* but low expression of p53 targets, relative to other cases^14^. Otherwise, the literature to date in regards to MDM4 function in liver cancer are limited to in vitro studies in HCC cell lines^22^.

In this paper, we explore the efficacy of inhibition of MDM4, in comparison to targeting MDM2, as a possible therapeutic strategy for HB. We assess the effects of the established MDM4 inhibitor NSC207895 (XI-006), which is known to inhibit gene expression of *MDM4* by physically binding its promoter and preventing transcription, induce apoptosis, and activate the p53 signaling pathway^23^, and the peptide dual *MDM2* and *MDM4* inhibitor ATSP-7041 in a range of in vitro and in vivo assays to show that targeting MDM4 in HB may be an effective treatment strategy for this disease.

## Results

### MDM4 is expressed in HB patient samples and cell lines

**Figure 1:**
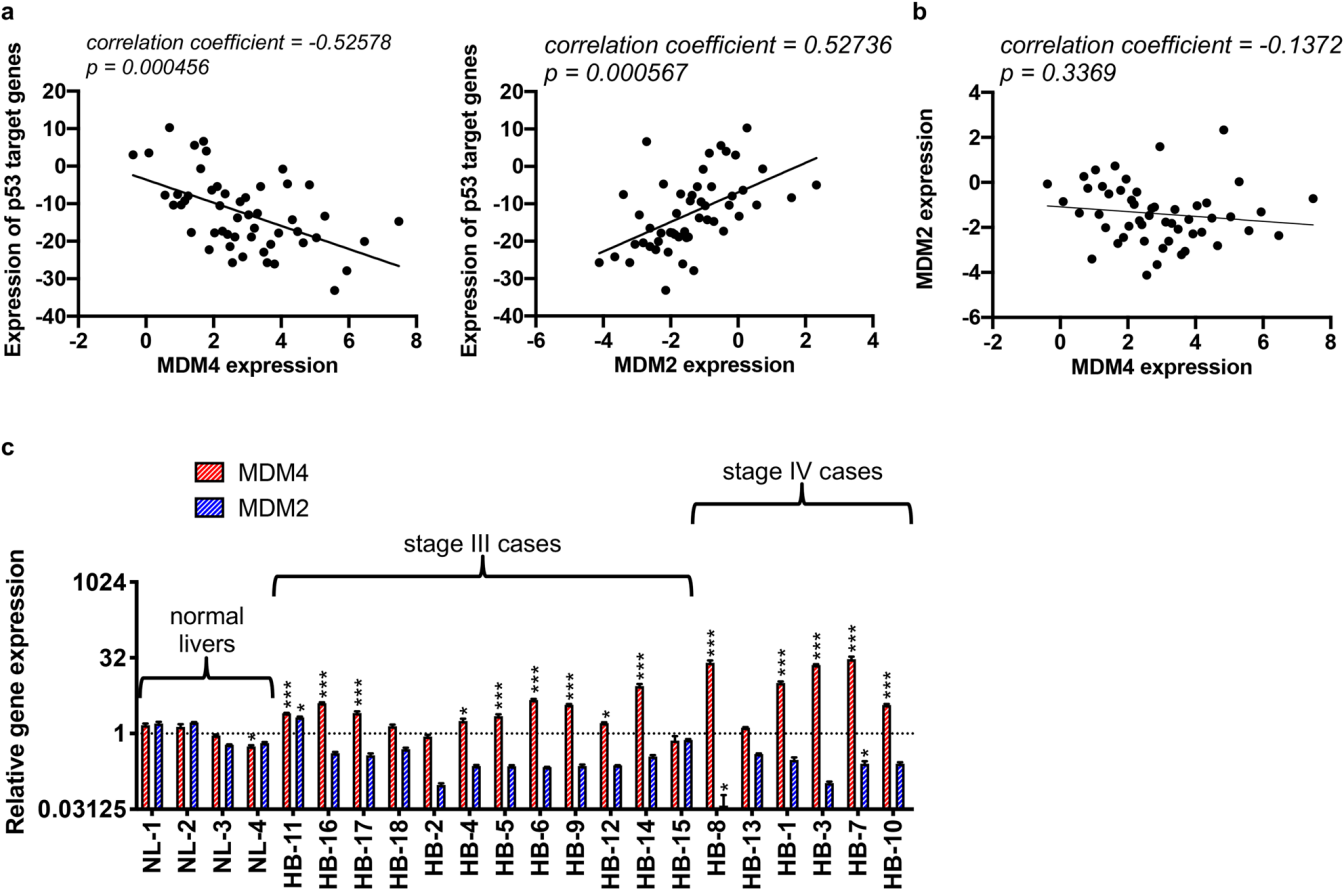
MDM4 expression is significantly elevated in HB patient samples and is correlated with increased expression of p53 target genes. (**a**) Dot plot representing expression profiles of *MDM4* and *MDM2* and normalized averages of the mRNA expression profiles of 22 select p53 target genes (*AEN*, *ALDH4A1*, *CDKN1A*, *DDB2*, *DUSP1*, *EDA2R*, *ESR1*, *FAS*, *FDXR*, *GADD45A*, *GADD45B*, *PANK1*, *PERP*, *PTCHD4*, *RPRM*, *RPS27L*, *RRM2B*, *SPATA18*, *TRIAP1*, *TRIM22*, *WRAP53*, *ZMAT3*), together with the optimal linear fit. (**b**) Dot plot representing expression of *MDM4* and *MDM2*, together with the optimal linear fit. (**c**) Bar graph representing normalized mRNA expression of *MDM2* and *MDM4* analyzed with qPCR experiments with 18 patient samples in comparison to four adjacent uninvolved liver samples. Y-axis shown with a log2 scale. Error bars represent SD. Data shown in b are representative of at least three independent experiments performed with three replicate wells each time. Student’s *t* test (two tailed) *P < 0.05, **P < 0.01, ***P < 0.001 representing comparison of each sample’s expression data to all expression data of uninvolved liver samples.

We first examined gene expression levels of *MDM4* and *MDM2* along with a panel of p53 target genes in an Affymetrix microarray dataset of 50 HB patients and six normal pediatric liver tissues described in a previous key publication^20^. These analyses showed that gene expression of 22 established p53 target genes used as a readout for p53 activity significantly correlated with both *MDM4* (*p* = *0.000456*) and *MDM2* (*p* = *0.000567*) expression in the tumor samples (Fig. 1a). Importantly, the expression profiles of these p53 target genes inversely correlated with *MDM4* expression (*correlation coefficient* = *-0.52578*) but directly correlated with *MDM2* expression (*correlation coefficient* = *0.52736*). Further analysis of this dataset did not reveal a correlation between *MDM2* and *MDM4* expression (Fig. 1b, *correlation coefficient* = *-0.1372*, *p* = *0.3369*) and risk group or presence of identified high risk molecular profiles^20,24^. In a second analysis of primary patient data, we examined levels of *MDM4* gene expression in a cohort of 18 primary HB samples from Children’s Oncology Group (COG) stage III (n = 12) and IV (n = 6) patients with quantitative reverse transcription polymerase chain reaction (qPCR) experiments in comparison to expression in four adjacent uninvolved liver samples. Of the 18 patients, there were eleven (61%) males and 7 (39%) females. The average age at diagnosis of these patients was 41.2 months. All patients presented were *TP53* wild-type. In 16 of the 18 patient samples, *MDM4* gene expression was higher than average expression in the uninvolved liver samples; in five samples expression was at least 5-fold higher and in four samples expression was at least 10-fold higher (Fig. 1c). Of note, all four samples with at least 10-fold higher expression were from stage IV patients. We also looked at *MDM2* mRNA expression as a comparison in these samples; *MDM2* expression was only elevated in one of the 18 patient samples compared to the uninvolved liver samples (Fig. 1c). In summary, this data clearly shows that *MDM4* gene expression is increased in primary HB tissues and that this correlates with downregulation of the p53 tumor suppressor signaling pathway, supporting further study of MDM4 as a target for disease therapy.

We also analyzed levels of MDM2 and MDM4 expression in the HB cell lines HepG2^25^, Huh-6^26^, HepT1^27^, and B6-2^28^ and the terminally differentiated liver cell line HepRG^29^ with qPCR experiments. Importantly, according to the literature, all of these cell lines are wild-type for the *TP53* gene^15,28,30^. The cell lines all showed clear *MDM4* gene expression (Supplementary Fig. 1a). Immunoblotting for MDM4 with the same five cell lines in comparison to the MCF-7 breast cancer cell line that is established to express high levels of MDM4 protein^31^ also showed that they all express MDM4 protein (Supplementary Fig. 1b,c). Taken together, these data showed clear gene and protein expression of MDM4 in HB cell lines.

### MDM4 inhibition leads to apoptosis of HB cell lines

**Figure 2:**
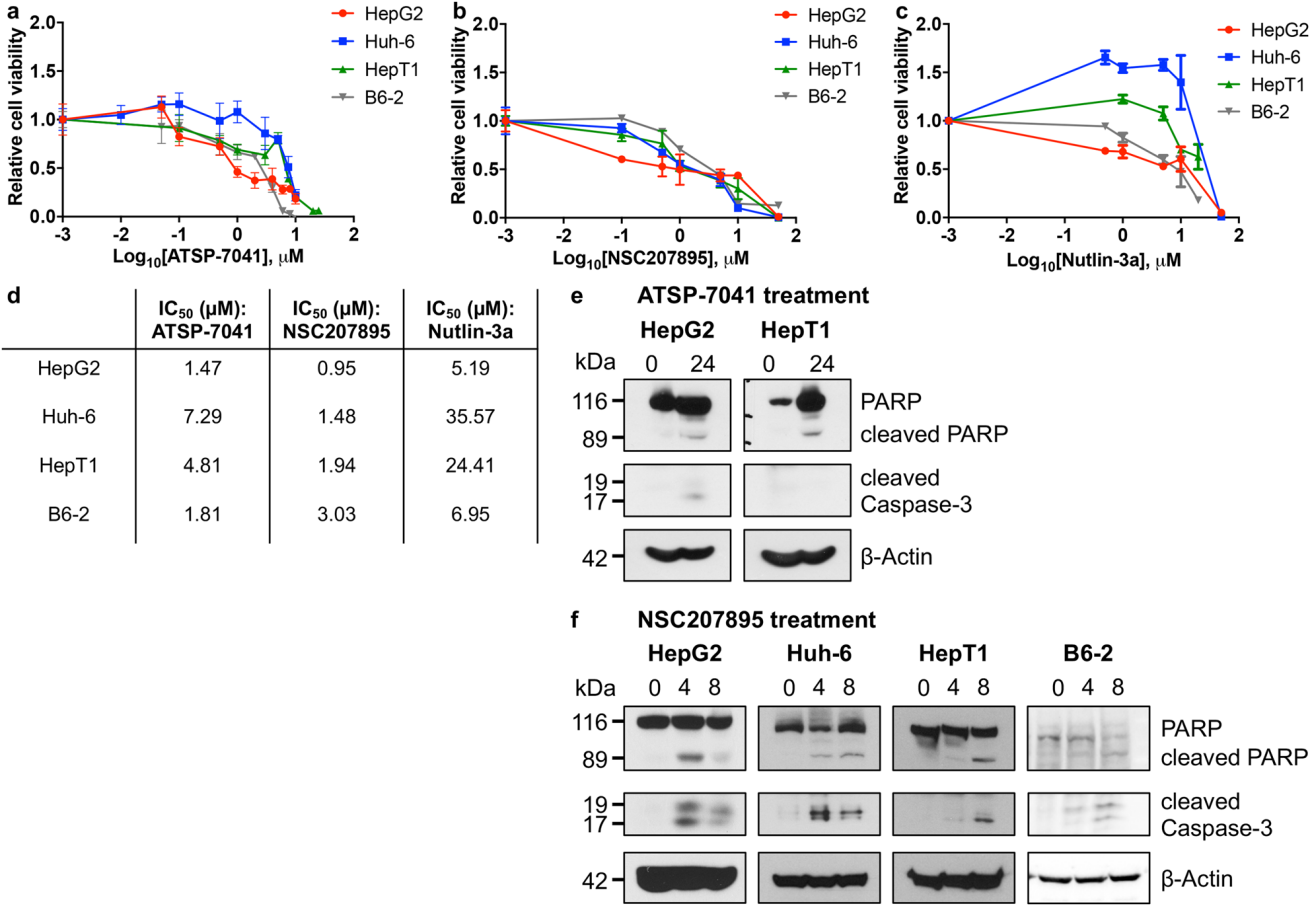
MDM4 inhibition with ATSP-7041 and NSC207895 lead to apoptosis of HB cell lines. (**a**) HepG2, Huh-6, HepT1, and B6-2 cells were exposed to varying indicated concentrations of ATSP-7041 for 48 h. MTT assays were performed at 48 h to assess viability. Error bars represent SD. Data shown are representative of at least three independent experiments performed with three replicate wells each time. (**b**) HepG2, Huh-6, HepT1, and B6-2 cells were exposed to varying indicated concentrations of NSC207895 for 48 h. MTT assays were performed at 48 h to asses viability. Error bars represent SD. Data shown are representative of at least three independent experiments performed with three replicate wells each time. (**c**) HepG2, Huh-6, HepT1, and B6-2 cells were exposed to varying indicated concentrations of Nutlin-3a for 48 h. MTT assays were performed at 48 h to asses viability. Error bars represent SD. Data shown are representative of at least three independent experiments performed with three replicate wells each time. (**d**) IC_50_ values determined in the assays shown in (**a**–**c**). **(e)** Protein lysis from cells treated with 10 μM ATSP-7041 for 24 h was compared to that from untreated cells (0 h). Immunoblotting for total PARP, PARP cleavage, and Caspase-3 cleavage was done to assess apoptosis. β-Actin immunoblotting was used as a loading control. Date shown are representative of at least three independent experiments. (**f**) Protein lysis from cells treated with 10 μM NSC207895 for 4 or 8 h was compared to that from untreated cells (0 h). Immunoblotting for total PARP, PARP cleavage, and Caspase-3 cleavage was done to assess apoptosis. β-Actin immunoblotting was used as a loading control. Data shown are representative of at least three independent experiments. Full length blots for data shown in e and f are presented in Supplementary Fig. 7.

We treated HB cell lines HepG2, Huh-6, HepT1, and B6-2 with NSC207895, which has been shown to inhibit gene expression of *MDM4*^23^; ATSP-7041, which is a stapled-peptide dual inhibitor of MDM2 and MDM4^32^; or the established MDM2 inhibitor Nutlin-3a^33^ and compared the responses of the cells to each agent with MTT assays. All four cell lines showed cytotoxic responses to inhibition of MDM4 with NSC207895 (IC_50_ 0.95 μM – 3.03 μM) or ATSP-7041 (IC_50_ 1.47 μM – 7.29 μM) (Fig. 2a–d). HepG2 cells were the most sensitive to both agents (IC_50_ 1.47 μM for ATSP-7041, 0.95 μM for NSC207895) while Huh-6 cells were the least sensitive to ATSP-7041 (7.29 μM) and B6-2 cells were the least sensitive to NSC207895 (3.03 μM). Nutlin-3a treatment also resulted in cell death but at much higher concentrations when compared to the other two inhibitors (IC_50_ 5.19 μM – 35.57 μM, Fig. 2c,d). We verified induction of apoptosis with ATSP-7041 or NSC207895 treatment by using immunoblotting assays to look for stimulation of Poly ADP ribose polymerase (PARP) and Caspase-3 cleavage with drug exposure. We detected clear evidence of apoptosis in HepG2 and HepT1 cells treated with ATSP-7041 and in HepG2, Huh-6, HepT1, and B6-2 cells treated with NSC207895, as shown by increases in PARP and Caspase-3 cleavage (Fig. 2e,f). In comparison, we observed much less PARP and Caspase-3 cleavage in HB cells treated with Nutlin-3a (Supplementary Fig. 2).

### MDM4 inhibition leads to upregulation of p53 activity and downstream signaling

**Figure 3:**
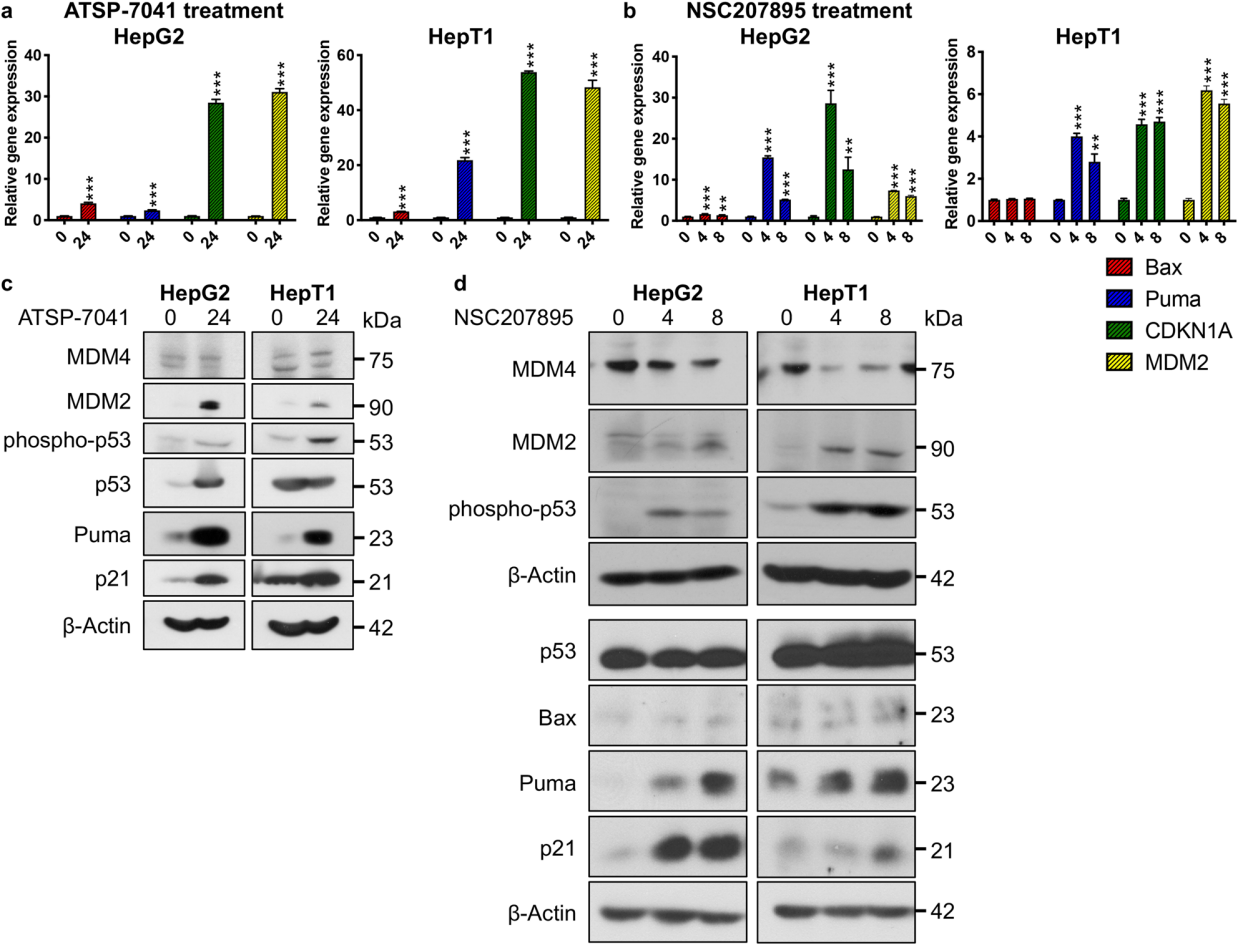
Acute MDM4 inhibition with ATSP-7041 and NSC207895 lead to upregulation of p53 activity and downstream signaling. (**a**) Bar graphs representing normalized mRNA expression of p53 targets *Bax*, *Puma*, *CDKN1A*, and *MDM2* analyzed with qPCR experiments. RNA extracted from HepG2 and HepT1 cells treated with 10 μM ATSP-7041 for 24 h was compared to that from untreated cells (0 h). Error bars represent SD. Data shown are representative of at least three independent experiments performed with three replicate wells each time. Student’s *t* test (two tailed) *P < 0.05, **P < 0.01, ***P < 0.001. (**b**) Bar graphs representing normalized mRNA expression of p53 targets *Bax*, *Puma*, *CDKN1A*, and *MDM2* analyzed with qPCR experiments. RNA extracted from HepG2 and HepT1 cells after treatment with 10 μM NSC207895 for 4 or 8 h was compared to that from untreated cells (0 h). Error bars represent SD. Data shown are representative of at least three independent experiments performed with three replicate wells each time. Student’s *t* test (two tailed) *P < 0.05, **P < 0.01, ***P < 0.001. (**c**) Protein lysis from cells treated with 10 μM ATSP-7041 for 24 h were compared to untreated cells (0 h). Immunoblotting was done with the indicated antibodies, including MDM4 (04–1555, Millipore). β-Actin immunoblotting was used as a loading control. Data shown are representative of at least three independent experiments. (**d**) Protein lysis from cells treated with 10 μM NSC207895 for 4 or 8 h were compared to untreated cells (0 h). Immunoblotting was done with the indicated antibodies, including MDM4 (04–1555, Millipore). β-Actin immunoblotting was used as a loading control. Data shown are representative of at least three independent experiments. Full length blots for data shown in c and d are presented in Supplementary Fig. 8.

We next investigated the effect of MDM4 inhibition with NSC207895 or ATSP-7041 treatment on p53 signaling in HB cell lines HepG2 and HepT1. We first examined gene expression of established p53 transcriptional targets *Bax*, *Puma*, *CDKN1A*, and *MDM2*. With ATSP-7041 treatment of HepG2 and HepT1, significant (*p* < *0.001*) increases in gene expression of all four targets (*Bax* 4.07 fold change (HepG2), 3.18 fold change (HepT1); *Puma* 2.38 fold change (HepG2), 21.85 fold change (HepT1); *CDKN1A* 28.49 fold change (HepG2), 53.81 fold change (HepT1); *MDM2* 31.07 fold change (HepG2), 48.3 fold change (HepT1)) were seen (Fig. 3a). Treatment with NSC207895 resulted in similar responsiveness of the p53 transcriptional targets with *Bax* showing the lowest response (1.63 (*p* < *0.001*) , 1.36 (*p* = *0.004*) fold change (HepG2); 1.05 (n.s.), 1.06 (n.s.) fold change (HepT1)) and *Puma* (15.45 (*p* < *0.001*), 5.15 (*p* < *0.001*) fold change (HepG2), 4.01 (*p* < *0.001*), 2.81 (*p* = *0.001*) fold change (HepT1)) , *CDKN1A* (28.63 (*p* < *0.001*), 12.5 (*p* = *0.0027*) fold change (HepG2), 4.58 (*p* < *0.001*), 4.71 *(p* < *0.001*) fold change (HepT1)) , and *MDM2* (7.39 (*p* < *0.001*), 6.01 (*p* < *0.001*) fold change (HepG2), 6.18 (*p* < *0.001*), 5.56 (*p* < *0.001*) fold change (HepT1)) mRNA expression showing the highest responses to treatment (Fig. 3b). Further, we looked at changes in protein expression of Bax, Puma, p21 (protein product of *CDKN1A*), MDM4, MDM2, phosphorylated p53 (phospho-p53, serine 15), and p53 in HepG2 and HepT1 cells exposed to ATSP-7041 and NSC207895. MDM4 protein expression was not reduced in HepG2 and HepT1 cells treated with ATSP-7041, which is consistent with the mechanism of action of this drug (Fig. 3c). Protein expression of MDM2, phospho-p53, Puma, and p21 were increased in both cell lines (Fig. 3c). Total p53 protein levels were also increased in HepG2 cells but not in HepT1 cells (Fig. 3c). With NS207895 treatment, MDM4 expression was reduced in both cell lines, supporting the established mechanism of action of this agent (Fig. 3d)^23^. Levels of p53 were unchanged among treated and untreated HepG2 samples but slightly increased in treated HepT1 cells (Fig. 3d, Supplementary Fig. 3). Finally, levels of p21, Bax, Puma, phospho-p53, and MDM2 were increased in both cell lines (Fig. 3d).

### Prolonged MDM4 inhibition slows proliferation of HB cell lines and activates p53 signaling

**Figure 4:**
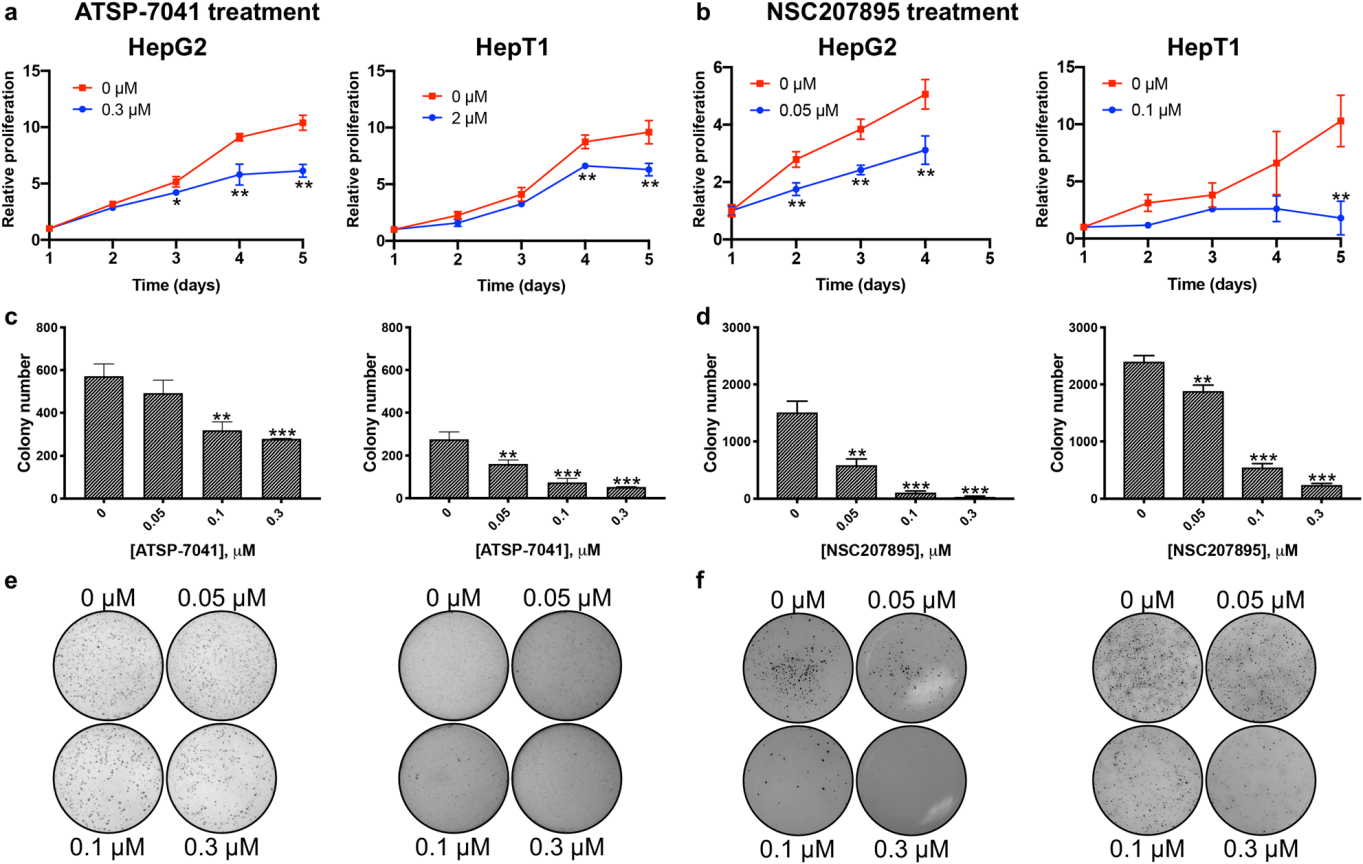
Inhibition of MDM4 leads to decreases in proliferation. (**a**) HepG2 and HepT1 cells were exposed to ATSP-7041 for 120 h and MTT assays were done at 24 h intervals to assess cell number. Data shown are representative of at least three independent experiments performed with three replicate wells each time. Error bars represent SD. Student’s *t* test *P < 0.05, **P < 0.01, ***P < 0.001. (**b**) HepG2 and HepT1 cells were exposed to NSC207895 for 96 or 120 h and MTT assays were done at 24 h intervals to assess cell number. Data shown are representative of at least three independent experiments performed with three replicate wells each time. Error bars represent SD. Student’s *t* test *P < 0.05, **P < 0.01, ***P < 0.001. (**c**, **e**) Cells were maintained in anchorage-independent conditions on a soft agar base with ATSP-7041 or vehicle for 2 to 3 weeks before staining with 500 μl of MTT solution for 4 h to visualize colonies. Images were captured by a VersaDoc Imaging System and colonies were counted with Quantity One software. Bar graphs in c represent colony numbers in indicated conditions. Data shown are representative of at least three independent experiments performed with three replicate wells each time. Error bars represent SD. Student’s *t* test *P < 0.05, **P < 0.01, ***P < 0.001. (**d**, **f**) Cells were maintained in anchorage-independent conditions on a soft agar base with NSC207895 or vehicle for 2 to 3 weeks before staining with 500 μl of MTT solution for 4 h to visualize colonies. Images were captured by a VersaDoc Imaging System and colonies were counted with Quantity One software. Bar graphs in d represent colony numbers in indicated conditions. Data shown are representative of at least three independent experiments performed with three replicate wells each time. Error bars represent SD. Student’s *t* test *P < 0.05, **P < 0.01, ***P < 0.001.

Because p53 controls pathways involved in cellular proliferation and senescence^13^, we investigated changes in cell proliferation that occurred with exposure to doses of ATSP-7041 and NSC207895 lower than the estimated IC_50_ concentrations (estimated percent viability of cells exposed to these doses for 48 h shown in Supplementary Tables 1, 2) for extended periods of time. First, we conducted MTT assays on consecutive days with HB cells exposed to sub-IC_50_ concentrations of the inhibitors (for ATSP-7041, 0.3 μM with HepG2, 2 μM with HepT1; for NSC207895, 0.05 μM with HepG2, 0.1 μM with HepT1). Both cell lines showed clear decreases in proliferation (*p* < *0.01*) , as compared to the untreated cells (Fig. 4a,b). To assess whether MDM4 inhibition affected anchorage-independent growth, we treated cells grown on soft agar with the same low doses of ATSP-7041 or NSC207895 (0.05–0.3 μM). With both cell lines, MDM4 inhibition led to a clear decrease in the anchorage-independent growth ability of the cells (*p* < *0.001*, Fig. 4c–f). Therefore, MDM4 inhibition blocked HB cell proliferation at low concentrations.

**Figure 5:**
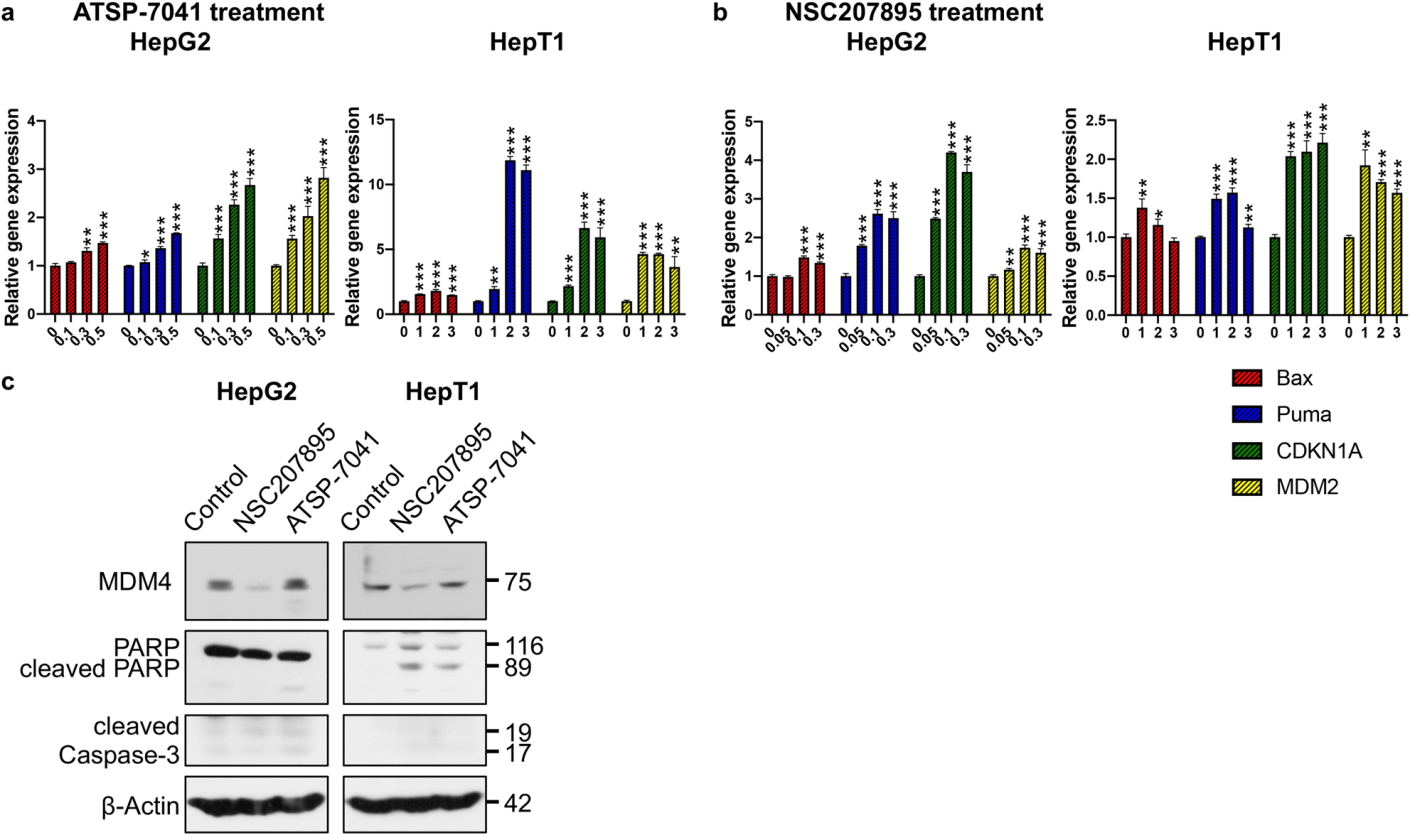
Prolonged MDM4 inhibition with ATSP-7041 and NSC207895 lead to upregulation of p53 activity and downstream signaling. (**a**) Bar graphs representing normalized mRNA expression of p53 targets *Bax*, *Puma*, *CDKN1A*, and *MDM2* analyzed with qPCR experiments. RNA extracted from HepG2 and HepT1 cells treated with the indicated concentrations of ATSP-7041 for 48 h was compared to that from untreated cells (0 h). Error bars represent SD. Data shown are representative of at least three independent experiments performed with three replicate wells each time. Student’s *t* test (two tailed) *P < 0.05, **P < 0.01, ***P < 0.001. (**b**) Bar graphs representing normalized mRNA expression of p53 targets *Bax*, *Puma*, *CDKN1A*, and *MDM2* analyzed with qPCR experiments. RNA extracted from HepG2 and HepT1 cells treated with the indicated concentrations of NSC207895 for 48 h was compared to that from untreated cells (0 h). Error bars represent SD. Data shown are representative of at least three independent experiments performed with three replicate wells each time. Student’s *t* test (two tailed) *P < 0.05, **P < 0.01, ***P < 0.001. (**c**) Protein lysis from cells treated with 0.5 μM ATSP-7041 or NSC207895 for 48 h were compared to untreated, control cells (0 h). Immunoblotting was done with the indicated antibodies, including MDM4 (04–1555, Millipore). β-Actin immunoblotting was used as a loading control. Data shown are representative of at least three independent experiments. Full length blots are presented in Supplementary Fig. 9.

In a second set of experiments, we examined changes in gene expression of p53 transcriptional targets *Bax*, *Puma*, *CDKN1A*, and *MDM2* with prolonged exposure to the MDM4 inhibitors with qPCR experiments. Treatment of HepG2 and HepT1 cells with low doses of ATSP-7041 (0.1–0.5 μM for HepG2, 1–3 μM for HepT1) for 48 h led to significant (*p* < *0.001*) changes in expression of *Bax* (1.47 fold change for HepG2, 1.8 fold change for HepT1), *Puma* (1.67 fold change for HepG2, 11.87 fold change for HepT1), *CDKN1A* (2.67 fold change for HepG2, 6.64 fold change for HepT1), and *MDM2* (2.82 fold change for HepG2, 4.63 fold change for HepT1) at 0.5 μM (HepG2) and 2 μM (HepT1) concentrations of inhibitor (Fig. 5a). Similarly, exposure of HepG2 and HepT1 cells with low doses of NSC207895 (0.05–0.3 μM for HepG2, 1–3 μM for HepT1) for 48 h led to significant (*p* < *0.01*) changes in expression of *Bax* (1.49 fold change for HepG2, 1.38 fold change for HepT1), *Puma* (2.62 fold change for HepG2, 1.57 fold change for HepT1), *CDKN1A* (4.2 fold change for HepG2, 2.1 fold change for HepT1), and *MDM2* (1.74 fold change for HepG2, 1.71 fold change for HepT1) at 0.1 μM (HepG2) and 1–2 μM (HepT1) concentrations of inhibitor (Fig. 5b).

We then verified inhibition of MDM4 protein expression at these lower doses of NSC207895 for 48 h to show that effects on p53 signaling were occurring in an MDM4-dependent manner. Immunoblotting experiments with cells treated with 0.5 μM NSC207895 and ATSP-7041 for 48 h showed clear lower expression of MDM4 protein with NSC207895 treatment but not with ATSP-7041 treatment (Fig. 5c), which is expected since ATSP-7041 does not affect expression levels of MDM4. We also examined induction of PARP and Caspase-3 cleavage at the same 0.5 μM dose of the drugs at 48 h to see if cell death was present under these conditions. With these experiments, we saw clear induction of PARP cleavage but no change in Caspase-3 cleavage (Fig. 5c), indicating that the cells do not fully undergo apoptosis. This supports our proliferation assays that indicate inhibition of proliferation with exposure of cells to low doses of the inhibitors for 48 h (Fig. 4a,b).

### Cytotoxic effects of NSC207895 are dependent on MDM4 and p53 signaling

**Figure 6:**
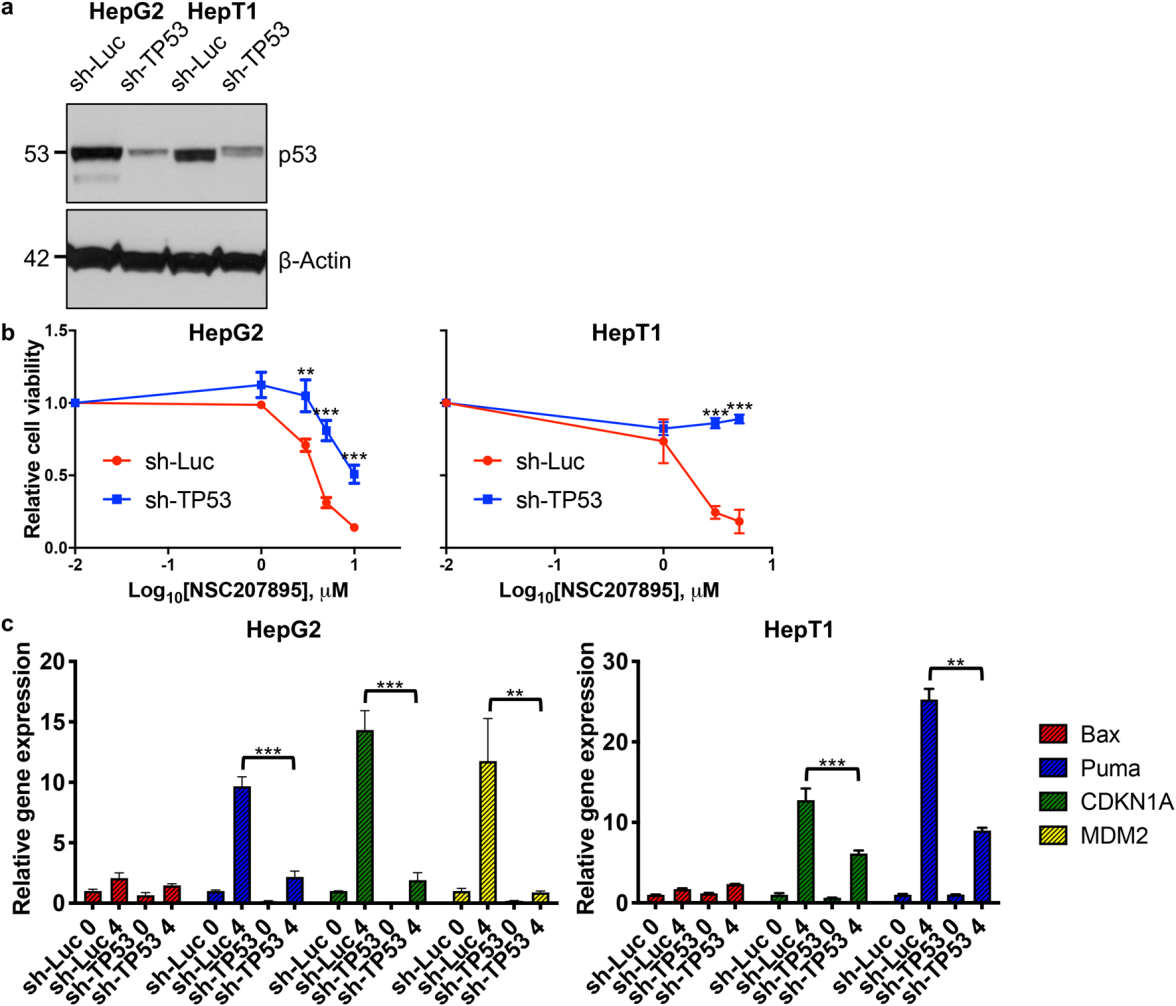
Cytotoxic effects of NSC207895 are dependent on p53 signaling. (**a**) Immunoblotting with HepG2 and HepT1 cells transduced with *sh-TP53* or control *sh-Luc* with an antibody recognizing p53. β-Actin immunoblotting was used as a loading control. Data shown are representative of at least three independent experiments. Full length blots for data shown in a are presented in Supplementary Fig. 10. (**b**) Cells transduced with *sh-TP53* or control *sh-Luc* were incubated with varying indicated concentrations of NSC207895 for 48 h. MTT assays were performed at 48 h to assess viability. (**c**) Bar graphs representing normalized mRNA expression of p53 targets *Bax*, *Puma*, *CDKN1A*, and *MDM2* analyzed with qPCR experiments. RNA was extracted from cells transduced with *sh-TP53* or control *sh-Luc* treated with 10 μM NSC207895 for 0 or 4 h. Expression in treated cells (4 h) was compared to that from untreated cells (0 h). Data shown in b and c are representative of at least three independent experiments performed with three replicate wells each time. Error bars represent SD. Student’s *t* test (two tailed) *P < 0.05, **P < 0.01, ***P < 0.001.

Since treatment of HB cells with NSC207895 led to increases in p53 signaling, we knocked-down *TP53* in HepG2 and HepT1 cells with short hairpin RNA (shRNA) targeting *TP53* and measured changes in the cytotoxic response to MDM4 inhibition with MTT assays. We reasoned that cells would become resistant to NSC207895 with decreased *TP53* expression. Sufficient knock-down of p53 was verified with immunoblotting assays with each cell line with *sh-TP53* compared to *sh-Luc* control (Fig. 6a). In both cell lines, knocking-down *TP53* led to significant (*p* < *0.001*) resistance to NSC207895 treatment, most markedly seen in HepT1 cells (IC_50_ value of 1.75 μM (*sh-Luc*) versus > 10 μM (*sh-TP53*)) (Fig. 6b, Supplementary Table 3). We conducted qPCR experiments for p53 targets *Bax*, *Puma*, *CDKN1A*, and *MDM2* to further verify that blocking p53 signaling with knock-down of *TP53* abrogated the ability of MDM4 inhibition to activate the p53 tumor suppressor signaling pathway. Indeed, with both HepG2 and HepT1 cells, expression of *CDKN1A* and *Puma* were significantly (*p* < *0.01*) less increased with NSC207895 treatment in the setting of knock-down of *TP53* (for *CDKN1A*, 14.33 fold change (*sh-luc*) versus 1.92 fold change (*sh-TP53*) (HepG2), 12.77 fold change (*sh-luc*) versus 6.14 fold change (*sh-TP53*) (HepT1); for *Puma*, 9.67 fold change (*sh-luc*) versus 2.18 fold change (*sh-TP53*) (HepG2), 25.27 fold change (*sh-luc*) versus 8.99 fold change (*sh-TP53*) (HepT1)) (Fig. 6c). In addition, *MDM2* was also significantly (*p* < *0.01*) less upregulated with NSC207895 treatment with *TP53* knock-down in HepG2 cells (11.76 fold change (*sh-luc*) versus 0.9 fold change (*sh-TP53*)) (Fig. 6c). Taken together, these experiments show that the effects of NSC207895 on viability and on tumor suppressor target gene expression are largely dependent on the p53 signaling pathway.

**Figure 7:**
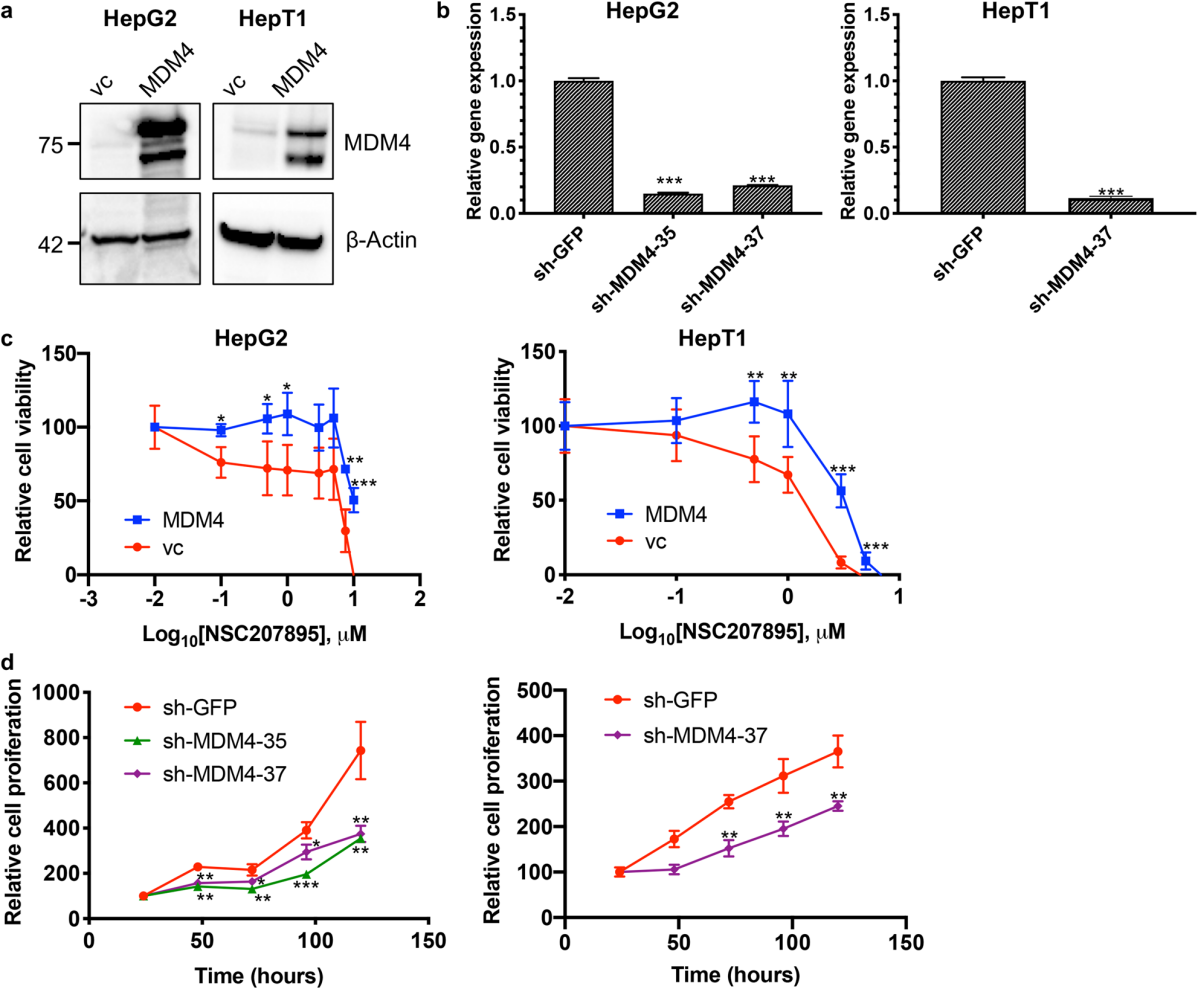
Cytotoxic effects of NSC207895 are dependent on MDM4. (**a**) Immunoblotting with HepG2 and HepT1 cells transduced with *MDM4* cDNA or vector control (vc) with an antibody recognizing MDM4 (04–1555, Millipore). β-Actin immunoblotting was used as a loading control. Data shown are representative of at least three independent experiments. Full length blots for data shown in a are presented in Supplementary Fig. 11. (**b**) Bar graphs representing normalized mRNA expression of *MDM4* analyzed with qPCR experiments. RNA extracted from HepG2 and HepT1 cells transduced with *sh-MDM4-35* or *sh-MDM4-37* was compared to that transduced with *sh-GFP*. (**c**) Cells transduced with *MDM4* cDNA or vc were incubated with varying concentrations of NSC207895 for 48 h. MTT assays were performed at 48 h to asses viability. (**d**) Cells transduced with *sh-MDM4* or control *sh-GFP* were grown for 120 h and MTT assays were done at 24 h intervals to assess cell number. Data shown in b, c, and d are representative of at least three independent experiments performed with three replicate wells each time. Error bars represent SD. Student’s *t* test *P < 0.05, **P < 0.01, ***P < 0.001.

In a second set of validation experiments, we manipulated levels of *MDM4* with knock-down and overexpression experiments. First, we tested the effects of *MDM4* overexpression in the setting of NSC207895 treatment. Using the HepG2 and HepT1 cell lines, we established *MDM4* overexpression with retroviral (HepT1) and lentiviral (HepG2) overexpression vectors (Fig. 7a). These cells were then treated with NSC207895, and their responses were compared to that of the vector control (vc) cells. The *MDM4* overexpressing cells showed significant (*p* < *0.001*) resistance to NSC207895-mediated cytotoxicity when compared to the vc cells (for HepG2, IC_50_ value of 5.3 μM (vc) versus > 10 μM (*MDM4*); for HepT1 cells, IC_50_ value of 1.19 μM (vc) versus 3.16 μM (*MDM4*)) (Fig. 7c, Supplementary Table 3). Second, we knocked-down expression of *MDM4* with lentiviral shRNA targeting *MDM4* to see if the observed changes in cells phenocopied their responses to NSC207895. We established HepG2 and HepT1 cell lines with clear knock-down of *MDM4* gene (74.5–88.4% knock-down, *p* < *0.001*) and protein expression (Fig. 7b, Supplementary Fig. 4). We conducted MTT assays on five consecutive days to measure changes in proliferation. Indeed, both HepG2 and HepT1 cells with knocked-down *MDM4* showed obvious (*p* < *0.05*) decreases in proliferation compared to control cells expressing *sh-GFP* (Fig. 7d), which was consistent with the effects of NSC207895 on proliferation. Specifically, HepG2 *sh-GFP* control cells have a growth rate approximately twofold higher and HepT1 *sh-GFP* control cells have a growth rate approximately 1.5-fold higher than the corresponding cells with knocked-down *MDM4* at the 120 h time point.

Finally, a previous study with NSC207895 proposed that the effects of the inhibitor were mediated through off-target effects on the genes *EDIL3*, *FLOT1*, *HEG1*, *UTRN*, *KIF20A*, *IDH1*, and *GPSM2* in studies of Ewing sarcoma and osteosarcoma cell lines^34^. We examined gene expression of these targets in HepG2 and HepT1 cells with qPCR experiments. To test whether the expression of these genes was dependent or independent of the MDM4-p53 axis, we examined changes in expression in HepG2 and HepT1 *sh-TP53* cells treated with NSC207895, as compared to control HepG2 and HepT1 *sh-Luc* cells. These experiments showed that only *UTRN* and *KIF20A* were changed significantly (*p* < 0.01) and in the same direction in all four samples tested (Supplementary Fig. 5). In the *sh-TP53* cells, however, *UTRN* was less changed, indicating that it was at least partially regulated by the MDM4-p53 signaling pathway. Only *KIF20A* expression was partially blocked in all four cell lines with NSC207895 treatment (HepG2 *sh-luc*, 0.80-fold change; HepG2 *sh-TP53*, 0.71-fold change; HepT1 *sh-luc*, 0.38-fold change; HepT1 *sh-TP53*, 0.4-fold change; *p* < 0.01) (Supplementary Fig. 5).

### In vivo efficacy of MDM4 inhibition in an orthotopic xenograft murine model of HB

**Figure 8:**
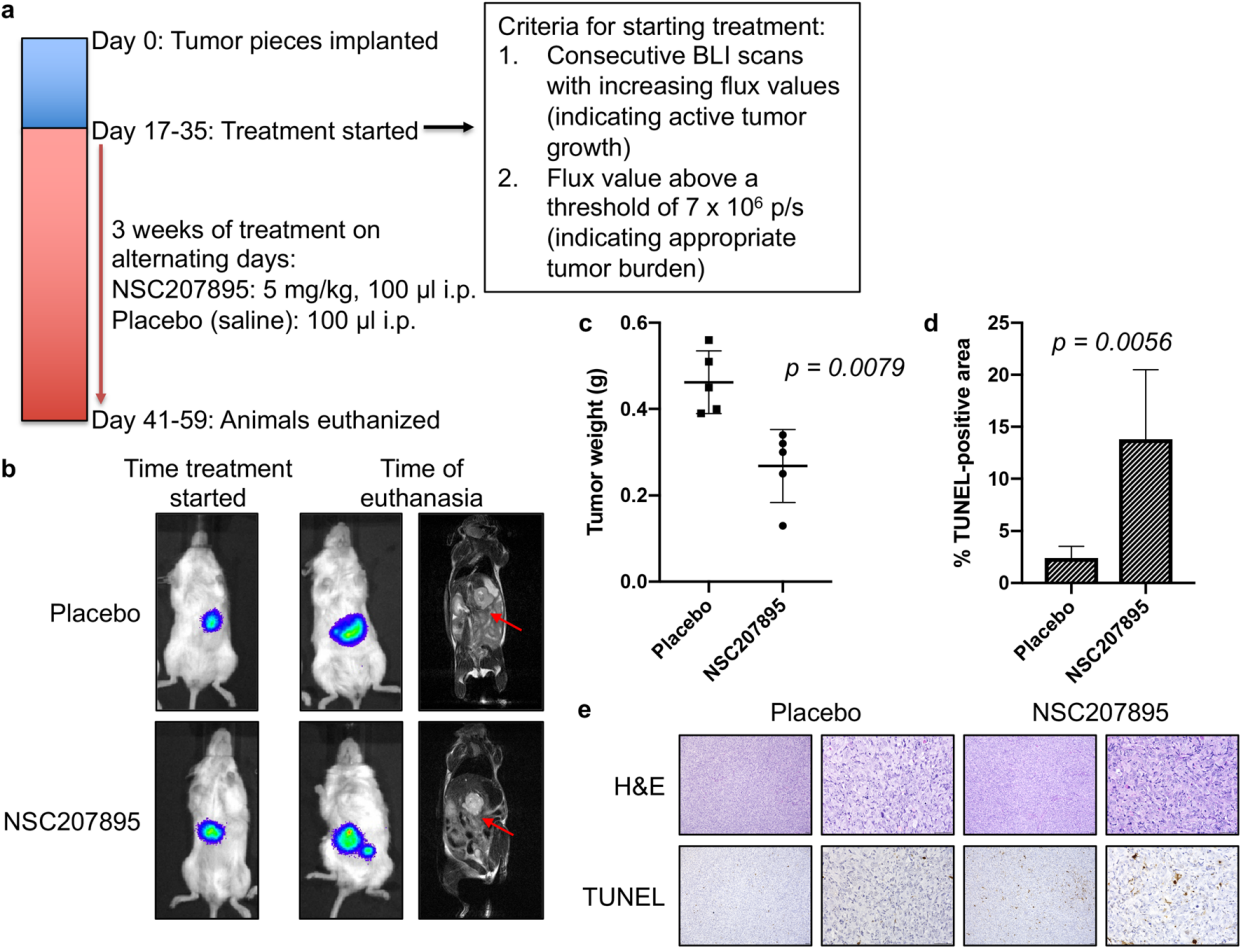
In vivo efficacy of MDM4 inhibition with an orthotopic xenograft murine model of HB. (**a**) Timeline of the animal study. (**b**) BLI and MRI images at early and late time points show progression of the HepT1-derived tumors in the placebo animals and animals treated with NSC207895. The cell line was stably transduced with *luciferase* to allow the use of BLI to monitor tumor growth in living animals. (**c**) Tumor weights of the placebo and NSC207895-treated animals at time of euthanasia. Kruskal Wallis test used to show significance. (**d**) Quantification of TUNEL staining of the tumors from the placebo and treatment animals. TUNEL-positive areas from images taken on a Keyence BZ-X710 All-in-One Fluorescence Microscope were quantified using the Keyence Hybrid Cell Count Analysis Application. Error bars represent SD. Student’s *t* test *P < 0.05, **P < 0.01, ***P < 0.001. (**e**) Representative pictures of H&E and TUNEL staining quantified in d. Scale bars represent 50 μm in 10X and 40X images.

To further validate our in vitro findings, we tested the effectiveness of MDM4 inhibition in a HepT1 orthotopic xenograft mouse model. All animal procedures used in this study were performed under an animal protocol approved by the Institutional Animal Care and Use Committee (IACUC) of Baylor College of Medicine (AN-6191) and were carried out in compliance with the ARRIVE guidelines. Intrahepatic tumors were generated using HepT1 cells transduced with *luciferase* to allow bioluminescence imaging (BLI) and treated with either NSC207895 or placebo. We treated animals at a dose of 5 mg/kg every other day for three weeks (Fig. 8a). Treatment was started when animals reached defined criteria (see methods). This occurred from 17 to 35 days after implantation for the 10 animals in the study.

With this study, weekly BLI, as well as magnetic resonance imaging (MRI) at a late time point, was conducted to monitor the in vivo growth of tumors and effects of MDM4 inhibition. When the animals were initially separated into treatment and placebo groups, BLI showed that the tumors were very comparable in size (Fig. 8b, Supplementary Fig. 6a), and BLI measurements during the course of the treatment period showed the growth of tumors with NSC207895 or placebo treatment (Supplementary Fig. 6a). At euthanasia, tumor weights were obtained. The HepT1-derived tumors treated with NSC207895 were significantly smaller than the tumors treated with vehicle (NSC207895-treated tumors were 58.01% the weight of placebo-treated tumors, *p* = *0.0079*) (Fig. 8c).

We then explored whether treatment with NSC207895 was leading to apoptosis by examining cell death in tissues with terminal deoxynucleotidyl transferase dUTP nick end labeling (TUNEL) assays. Tissues from animals treated with NSC207895 showed clear evidence of apoptosis, as indicated by TUNEL staining in the tumor, and quantification of this staining showed a significant (*p* = *0.0056*) difference between the treatment and placebo groups (Fig. 8d,e). We also examined inhibition of proliferation in these tissues with Ki67 staining (Supplementary Fig. 7c), and although the NSC207895 treated tumors showed a decrease in percent Ki67 positive area compared to the placebo treated tumors (Supplementary Fig. 6b), this did not reach significance. Taken together, this data showed that inhibition of MDM4 shows strong efficacy in an in vivo model of HB. Importantly, treatment with NSC207895 had minimal effects on normal liver tissue in the animals, as shown by TUNEL staining of the neighboring normal mouse liver that appears consistent between drug and placebo treated animals (Supplementary Fig. 6d).

## Discussion

The idea of targeting the negative regulators of p53 in order to reactivate activity of the tumor suppressor protein in cancer cells is a strategy that has been the focus of a substantial amount of research since the identification of MDM2 inhibitors^33^. This significant pharmaceutical effort to target MDM2 has led to nine current agents in clinical development at various stages^35^. Small molecule inhibitors of MDM2 have shown anti-tumor efficacy in preclinical studies but disappointing results in clinical trials with many patients showing toxicities that were not seen in prior mouse studies, including hematological effects, such as thrombocytopenia leading to hemorrhage, and increased incidence of *TP53* mutations with prolonged exposure^35–37^. Blocking MDM4 regulation of p53 is a more uncharted strategy with no agents specifically targeting only this protein in clinical development. Importantly, the stapled peptide dual inhibitor of MDM2 and MDM4, ALRN-6924, is currently being tested in four active clinical trials for a range of pediatric and adult malignancies, and this peptide shows an improved safety profile from the previous MDM2 small molecule inhibitors^38^.

Cancers that are characterized by overexpression of MDM4 are particularly likely to respond to such therapies. Many solid tumors, including gliomas, soft tissue sarcomas, head and neck squamous carcinomas, retinoblastomas, melanomas, breast cancers, and HBs show amplification of the *MDM4* gene^12,19^. A previous study of 56 HB tumors identified gains of the 1q chromosome as the most frequent allelic imbalance in HB (28 of 56 tumors)^19^. Further, genomic amplification limited to the 1q32.1 region in which the *MDM4* gene is located was noted in four tumors^19^. A second study of 46 HB tumors detected 1q gains in 34% of cases with these gains more frequently identified in high-risk cases^20^. In the present study, we examined levels of MDM4 expression in two cohorts of HB patient samples and showed that gene expression was increased in primary HB tissues, with the highest expression of *MDM4* seen in the stage IV patients (Fig. 1). In addition, this work showed that *MDM4* gene expression in patient samples significantly correlates with decreased expression of p53 target genes (Fig. 1a), supporting a strong relationship between *MDM4* overexpression and p53 tumor suppressor pathway inhibition. In contrast, *MDM2* gene expression increased with increased expression of p53 target genes (Fig. 1a), indicating that MDM2 is more a marker of p53 tumor suppressor activity and may not serve as the key regulator of p53 activity in HB. Interestingly, when looking at our HB cell lines, we found a discrepancy in mRNA versus protein expression of MDM4 (Supplementary Fig. 1). We feel that this is due to well described post-translational modification of the MDM4 protein, including phosphorylation/dephosphorylation and ubiquitination^39^. In most of our immunoblotting experiments, we visualize two bands corresponding to MDM4, which we believe represent these post-translationally modified proteins or to several isoforms that are very close in size. Importantly, these bands are present with two different MDM4 primary antibodies and also in an MCF-7 control cell lysate that represents a cell line that highly expresses MDM4.

We then explored the efficacy of the chemical inhibitor NSC207895 in comparison to effects of the established MDM2 inhibitor, Nutlin-3a, and the stapled peptide dual inhibitor of MDM2 and MDM4, ATSP-7041. NSC207895 was identified in a promoter-based screen for compounds that block *MDM4* gene expression^23^ and has also been suggested to function as a DNA damaging agent^40^ while Nutlin-3a was the most potent agent found in a screen of a chemical library for compounds that bind to the MDM2 protein and inhibit its interaction with p53^33^. ATSP-7041 is a progenitor of the clinical compound ALRN-6924 that specifically binds to and inhibits both major p53 regulators, MDM2 and MDM4^32^. All three inhibitors have been well characterized to upregulate transcription of p53 targets including *Bax*, *Puma*, *CDKN1A*, and *MDM2* in *TP53* wild-type cells^23,32,33,41,42^. Treatment of cell lines with ATSP-7041 and NSC207895 led to slowing of proliferation at low doses (Fig. 4) and apoptosis at higher doses (Fig. 2). Two prior studies in glioma and melanoma similarly showed anti-oncogenic effects with inhibition of MDM4 with shRNA targeting *MDM4* or with SAH-p53-8, a stapled p53 helix that binds to MDM4 to block its interaction with endogenous, wild-type p53^43,44^. Notably, our IC_50_ values for cytotoxicity with NSC207895 and ATSP-7041 treatment were as much as 24-fold lower than for MDM2 inhibition with Nutlin-3a (Fig. 2d). Subsequent efforts to optimize Nutlin-3a led to the creation of the second-generation MDM2 inhibitor RG7112^45^ and the third-generation inhibitor RG7388^46^, and it is possible that treatment of HB cells with these more potent agents may lead to more effectiveness; however, the enthusiasm for targeting MDM2 in these patients is lessened by the known toxicities that have been described in phase I trials. It is also notable that sensitivity to NSC207895 and ATSP-7041 may be related to expression of MDM4 in that HepG2 was the most sensitive to both agents (Fig. 2d) and had the lowest levels of protein expression of MDM4 (Supplementary Fig. 1b,c). At the same time, HepT1 cells had the highest protein expression of MDM4 (Supplementary Fig. 1b,c) and yet were not the most resistant to either drug (Fig. 2d). It is likely that simple readouts of MDM4 gene and protein expression are not sufficient to predict sensitivity to these drugs that affect a complex cascade of tumor suppressor signaling.

To verify that the effects of NSC207895 and ATSP-7041 were occurring predominantly through reactivation of p53 downstream signaling, we measured changes in expression of p53 targets Bax, Puma, *CDKN1A*/p21, and MDM2 (Figs. 3, 5). Increases in gene and protein expression of these targets were seen in both cell lines examined. Interestingly, the two inhibitors showed their maximal effects on gene expression of p53 targets at different time points, NSC207895 at shorter time points of 4 and 8 h and ATSP-7041 at a longer time point of 24 h. We hypothesize that this is due to the different physical nature of the agents as NSC207895 is a small chemical compound and ATSP-7041 is a stapled peptide inhibitor. Further, levels of p53 protein were stable in HepG2 cells treated with NSC207895 (Fig. 3d, Supplementary Fig. 3), showing a unique characteristic of MDM4 inhibition as compared to ubiquitination effects associated with MDM2. On its own, MDM4 acts only to sequester the transcription factor activity of p53 without leading to its degradation^47^. Since the cells are not experiencing additional genotoxic stress with NSC207895, p53 degradation is not affected^47^. We anticipate that if we introduced genotoxic stress in combination with NSC207895 treatment, p53 protein levels would measurably increase. In the setting of NSC207895 treatment, inhibition of MDM4 only releases p53 from this inhibitory interaction without changing its protein levels. A key paper that defined the functional relationship between MDM2 and MDM4 similarly showed that knocking-down *MDM4* did not affect protein expression of p53 unless MDM2 was also manipulated^48^.

Because of a previous report that suggested that the chemical inhibitor NSC207895 acts on alternative targets in an MDM4/p53-independent manner^34^, we further validated the specificity of the observed effects by knocking-down *TP53* or overexpressing *MDM4* to change the responsiveness of cells to the agent. HepG2 cells with *sh-TP53* showed a shift in IC_50_ value from 4.01 μM to 9.94 μM (Fig. 6b), indicating that residual *p53* expression is enough to kill cells with MDM4 inhibition or, alternatively, NSC207895 is acting in a p53-independent manner in these cells in the absence of *p53*. Notably, HepT1 cells expressing *sh-TP53* showed resistance to the inhibitor with greater than 50% survival exhibited at doses exceeding the estimated IC_50_ value (Fig. 6b). Thus, the observed cytotoxicity predominantly depends on the MDM4-p53 axis. However, we cannot completely rule out minor off-target effects contributing to the observed consequences of exposure. These differences observed between cell lines is likely due to innate characteristics of these cells and their unique MDM4 and p53 signaling pathways. In the aforementioned study, seven genes were suggested to be alternative targets of NSC207895 that mediate the observed effects on cell survival, cell cycle arrest, and senescence, *EDIL3*, *FLOT1*, *HEG1*, *UTRN*, *KIF20A*, *IDH1*, and *GPSM2*. We examined effects of treatment with the agent on these targets in HepG2 and HepT1 cells with concomitant knock-down of *TP53*, in comparison to cells expressing control *sh-Luc*. In these experiments, we found that only *KIF20A* expression showed a consistent decrease in cells treated with NSC207895 no matter the levels of *TP53* (Supplementary Fig. 5) and may contribute to the observed effects of the agent. The KIF20A protein is necessary for normal cleavage furrow ingression and cytokinesis during cell division^49^ and has been identified as an oncogene in pancreatic cancer, gastric cancer, glioma, cervical squamous cell carcinoma, lung adenocarcinoma, and HCC^49–57^. In addition, a study showed that *KIF20A* knock-down in Huh-6 HB cells inhibited proliferation without inducing apoptosis^58^. Therefore, although it is clear that our results are predominantly dependent on inhibition of MDM4, it is possible that an effect on KIF20A is also contributing to the observed anti-tumor effects.

To date, much HB preclinical research has utilized subcutaneous models that do not recapitulate the tumor microenvironment of the liver that is so important to the phenotype of HB tumors. This paper represents the first instance that the HepT1 orthotopic xenograft mouse model has been described. Importantly, NSC207895 showed in vivo efficacy with this unique orthotopic xenograft model of HB (Fig. 8, Supplementary Fig. 6). Tissue samples from tumors treated with the inhibitor displayed cell death that was generally absent from placebo-treated tumors (Fig. 8d,e). Quantification of this cell death showed a statistically significant difference between placebo and drug treated tumors (Fig. 8d). Similarly, inhibition of MDM4 with SAH-p53-8, an early progenitor of ATSP-7041 and ALRN-6924, showed efficacy in an animal model of melanoma^43^. Interestingly, overexpression of *MDM4* in glioma and melanoma mouse models led to enhanced tumorigenesis and blocked the effectiveness of chemotherapies^43,44^, all suggesting that MDM4 may be oncogenic and lead to chemotherapy resistance in vivo.

Taken together, our in vitro and in vivo data from this study strongly supports further development and testing of agents to specifically inhibit MDM4 to reactivate the p53 tumor suppressor pathway. Currently, no targeted agents are approved for adjuvant use with HB patients. Only three cell lines are commercially available, making it difficult to obtain the preclinical data necessary to move agents into clinical trials. Importantly, in all in vitro and in vivo experiments in this study, ATSP-7041 and NSC207895 showed anti-tumor effects by reactivating p53 signaling leading to decreased cellular proliferation and apoptosis. Overall, this study supports further examination of MDM4 inhibition as a clinical strategy for high-risk HB patients. The key to this therapeutic approach is the mutation status of *TP53*, in that all malignancies with wild type *TP53* may respond to MDM4 targeted agents. Therefore, although this study only deals directly with HB tumors, the conclusions may be applicable to many other cancers, including a majority of pediatric cancers and liver cancers. These results support the first clinical trial of ALRN-6924 in pediatric patients (ClinicalTrials.gov Identifier: NCT03654716), which specifically includes enrollment of patients with histological diagnosis of HB and wild-type *TP53*.

## Methods

### Patient samples

The patient samples employed in these studies were collected from patients after informed consent from either the patients or their guardians was obtained via an Institutional Review Board-approved tissue collection protocol. All experiments on patient tissue samples were performed in compliance with the Helsinki Declaration and were approved by the Baylor College of Medicine Institutional Review Board.

### Gene expression of patient samples

For the cohort of 50 HB tumors and six normal pediatric liver tissues (Fig. 1a), RNA was extracted, mRNA expression was profiled by Affymetrix microarrays, and data was analyzed as described previously^20^. Twenty-two genes established to be transcriptional targets of p53 (*AEN*, *ALDH4A1*, *CDKN1A*, *DDB2*, *DUSP1*, *EDA2R*, *ESR1*, *FAS*, *FDXR*, *GADD45A*, *GADD45B*, *PANK1*, *PERP*, *PTCHD4*, *RPRM*, *RPS27L*, *RRM2B*, *SPATA18*, *TRIAP1*, *TRIM22*, *WRAP53*, *ZMAT3*), including 17 used as a readout for p53 activity in a key publication describing extensive profiling of HCC^14^, were further analyzed as a readout of p53 activity. For genes that had multiple probesets, the probeset that correlated most strongly with *TP53* expression was used. Normalized expression profiles of each gene were then summed to get a p53-activity inference, and this inference was correlated with *MDM4* and *MDM2*. *MDM4* and *MDM2* were also correlated with each other. Correlation was quantified with a standard correlation coefficient, and p values were corrected for the number of probesets tested per gene. For the cohort of 18 HB tumors and four adjacent uninvolved liver samples (Fig. 1b), RNA from frozen hepatic tumor and adjacent normal liver samples was isolated using the *mirVana* miRNA isolation kit (Ambion, Austin, TX, USA). Samples were treated with DNase 1 and eluted in nuclease-free water. RNA purity and quantity were determined using a spectrophotometer measuring absorbance at 260/280 nm. cDNA was generated from total RNA with the SuperScript III First-Strand Synthesis System for RT-PCR (Invitrogen). Taqman qPCR was done with TaqMan Universal Master Mix II (Applied Biosystems, Foster, CA, USA) and with the following primers (Applied Biosystems): *MDM4* (Hs00910358_s1) and *MDM2* (Hs00242813_m1). *GAPDH* (Hs02758991_g1) was used as an internal control in all qPCR experiments. All experiments were run on a StepOnePlus Real-Time PCR System (Applied Biosystems). Samples were normalized first to *GAPDH* and then to an average of the uninvolved liver samples using the ΔΔC_T_ method.

### Cells and culture conditions

The HepG2, Huh-6, and HepRG cell lines used in this study were commercially acquired (HepG2, HepRG: American Type Culture Collection (ATCC), Manassas, VA, USA; Huh-6: Riken Cell Bank, Japan). The HepT1 cell line was generously provided by Dr. Stefano Cairo (XenTech, France). The B6-2 cell line was generously provided by Dr. Karl-Dimiter Bissig (Baylor College of Medicine, Houston, TX, USA). The MCF-7 cell line was generously provided by Dr. Jianhua Yang (Baylor College of Medicine, Houston, TX, USA). Cell lines were grown at 37 °C in 5% CO_2_ in Eagle’s Minimum Essential Medium (EMEM, Lonza, Allendale, NJ, USA) supplemented with 10% heat-inactivated fetal bovine serum (FBS, SAFC Biosciences, Lenexa, KS, USA), 2 mM glutamine (Invitrogen, Carlsbad, CA, USA), and 100 units/ml streptomycin/penicillin (Invitrogen).

### Inhibitors

NSC207895 was purchased from EMD Millipore (444,158, Billerica, MA, USA). ATSP-7041 was generously provided by Aileron Therapeutics. Nutlin-3a was purchased from Sigma-Aldrich (N6287, St. Louis, MO, USA). All three were provided as powders and resuspended in dimethyl sulfoxide (DMSO) to generate 10 mM stock solutions. For the in vitro experiments, all three were diluted in media to the appropriate concentrations prior to use. For the in vivo experiments, NSC207895 was diluted in sterile saline to the appropriate concentration prior to use.

### Proliferation and cell viability assays to assess effects of NSC207895, ATSP-7041, Nutlin-3a, and MDM4 knock-down

To examine cytotoxicity, HB cell lines were plated in 96-well plates (5,000 cells/well) and allowed to settle for 24 h. NSC207895, ATSP-7041, or Nutlin-3a was added to each well at the indicated concentrations. 3-(4,5-dimethylthiazol-2-yl)-2,5-diphenyltetrazolium bromide (MTT) assays were performed after 48 h exposure to the agents by replacing the media in each well with 9% MTT (5 mg/ml) in media. After a 2 h incubation at 37 °C, 85 μl of MTT/media solution was removed from each well and 50 μl of DMSO was added. The plate was then read at 550 nm in a multimode plate reader (Beckman Coulter, Brea, CA, USA) within 10 min. IC_50_ values were estimated using GraphPad Prism (version 7.0a, GraphPad Software, Inc., La Jolla, CA, USA) with a nonlinear regression model of log(inhibitor) vs. normalized response – variable slope. To examine proliferation, HB cell lines were plated in 96-well plates (1,000 cells/well) and allowed to settle for 24 h. ATSP-7041 or NSC207895 was added to each well at the indicated concentrations. MTT assays were performed on consecutive days after exposure to the agents as described above. Effects of *MDM4* knock-down on proliferation were examined in a similar way with *sh-MDM4* or *sh-GFP* cells plated in 96-well plates and MTT assays performed on consecutive days beginning 24 h after plating. To examine anchorage-independent growth, HB cell lines were seeded in 6-well plates on a soft agar base. Briefly, 2 ml of a mixture of sterile 0.5% (w/v) agar (Becton, Dickinson and Company, Franklin Lakes, NJ, USA) in complete EMEM media was made and added to the bottom of each well. The top layer was made by mixing 1.5 ml of a mixture of sterile 0.3% (w/v) agar in complete EMEM media with 10,000 cells per well. After allowing cells to settle for 24 h, the indicated concentrations of ATSP-7041 or NSC207895 were added to each well. Cells were then maintained in culture for 2 to 3 weeks before staining with 500 μl of MTT solution for 4 h. Images were then captured with a VersaDoc Imaging System (Bio-Rad Laboratories, Inc., Hercules, CA, USA) and colonies were counted with Quantity One software (Bio-Rad Laboratories, Inc.).

### Quantitative RT-PCR with HB cell line samples

HB cell lines were plated (1 × 10^6^ cells per 6-cm plate) and cultured for 48 h directly prior to RNA collection. For experiments to examine the effects of NSC207895 or ATSP-7041, each agent was added while cells were in logarithmic growth phase. For the experiments shown in Figs. 3a,b and 6c with shorter time points, 10 μM NSC207895 or ATSP-7041 was added to each plate and cells were collected at 0, 4, and 8 h (NSC207895) or 0 and 24 h (ATSP-7041). For the experiments shown in Fig. 5a,b with longer time points, 0.1–3 μM ATSP-7041 or 0.05–3 μM NSC207895 was added to each plate and cells were collected at 0 and 48 h. RNA was extracted from cells with the Direct-zol RNA MiniPrep Kit (Zymo Research, Irvine, CA, USA). RNA purity and quantity were determined using a spectrophotometer measuring absorbance at 260/280 nm. cDNA was generated from total RNA with the qScript cDNA SuperMix (Quanta Biosciences, Gaithersburg, MD, USA). Taqman qPCR was done with TaqMan Universal Master Mix II (Applied Biosystems, Foster, CA, USA) and with the following primers (Applied Biosystems): *MDM4* (Hs00910358_s1), *MDM2* (Hs00242813_m1), *TP53* (Hs01034249_m1), *Bax* (Hs00180269), *Puma* (Hs00248075_m1), *CDKN1A* (Hs00355782_m1), *HEG1* (Hs00393516_m1), *FLOT1* (Hs00195134), *UTRN* (Hs01127967_m1), *EDIL3* (Hs00964112_m1), *IDH1* (Hs01855675_s1), *KIF20A* (Hs00993573_m1), and *GPSM2* (Hs00203271_m1). *GAPDH* (Hs02758991_g1) was used as an internal control in all qPCR experiments. All experiments were run on a StepOnePlus Real-Time PCR System (Applied Biosystems). Samples were normalized first to *GAPDH* and then to control samples as described in each experiment using the ΔΔC_T_ method.

### Immunoblotting with HB cell lines

HB cell lines were plated (2 × 10^6^ cells per 10-cm plate) and cultured for 48 h directly prior to protein collection. For experiments to examine the effects of ATSP-7041, NSC207895, and Nutlin-3a, the agent was added while cells were in logarithmic growth phase. For the experiments shown in Figs. 2e,f, 3c,d and Supplementary Fig. 2 with shorter time points, 10 μM ATSP-7041 or NSC207895 or 20 μM Nutlin-3a were added to each plate and cells were collected at 0, 4, and 8 h (NSC207895, Nutlin-3a) or 24 h (ATSP-7041). For the experiments shown in Fig. 5c with longer time points, 0.5 μM NSC207895 or ATSP-7041 were added to each plate and cells were collected at 0 and 24 h. Cell proteins were lysed with radioimmunoprecipitation assay (RIPA) buffer with protease inhibitors. Protein concentration was determined with Bio-Rad Protein Assay Dye Reagent Concentrate (Bio-Rad Laboratories, Inc.). One hundred μg protein was mixed with Novex NuPAGE LDS Sample Buffer (4X) and Novex Bolt Sample Reducing Agent (10X) (Life Technologies, Carlsbad, CA, USA), boiled, resolved by sodium dodecyl sulfate polyacrylamide gel electrophoresis (SDS-PAGE), and transferred to nitrocellulose membranes with an iBlot 2 apparatus (Invitrogen). Membranes were blocked with phosphate buffered saline (PBS) with 10% Tween and 5% non-fat milk for 1 h at room temperature with rocking. Membranes were then incubated at 4 °C overnight with rocking with primary antibodies for MDM4 (1:1000, NBP1-28,862, Novus Biologicals, Littleton, CO, USA (Supplementary Fig. 1c) or 1:2000, 04–1555 (clone 8C6), Millipore Sigma, Danvers, MA, USA (Figs. 3c,d, 5c, 7a, Supplementary Fig. 4)), PARP (1:1000, 9532, Cell Signaling Technology, Danvers, MA, USA), Caspase-3 (1:500, 14,220, Cell Signaling), MDM2 (1:500, sc-965, Santa Cruz Biotechnology, Inc., Dallas, TX, USA), p53 (1:1000, sc-126, Santa Cruz Biotechnology, Inc.), phospho-p53 (serine 15, 1:1000, 9286, Cell Signaling), Bax (1:1000, sc-493, Santa Cruz Biotechnology), Puma (1:500, D30C10, Cell Signaling), p21 (1:1000, SX118, Santa Cruz Biotechnology), and β-Actin (1:30,000, A2228, Sigma-Aldrich). The membranes were then incubated with horseradish peroxidase (HRP)-conjugated anti-mouse (7076S) or anti-rabbit (7074S) secondary antibodies (Cell Signaling). Immunoblot band densities were determined with ImageJ (v1.46r, NIH, USA) as previously described^59^. Relative intensity levels were determined by dividing the band intensity of the total protein by the intensity of the loading control protein (β-Actin).

### shRNA and overexpression constructs

The lentiviral psi-LVRU6MP-GFP (*sh-GFP*), psi-LVRU6MP-MDM4-35 (*sh-MDM4-35*), and psi-LVRU6MP-MDM4-37 (*sh-MDM4-37*) constructs were purchased from Genecopoeia (Rockville, MD, USA). The pLSLPw-Luc (*sh-Luc*) and pLSLpw-TP53 (*sh-TP53*) constructs were generously provided by A.V. Budanov as described^60^. Both *sh-Luc* and *sh-TP53* sequences were cloned into the lentiviral vector for use in the *sh-TP53* experiments. For the HepT1 *MDM4* overexpression experiment, the pBABE-MDM4 retroviral construct was generated by cloning the cDNA sequence of MDM4 into the pBABE vector. The empty pBABE vector (pBABE-vector control (vc)) was used as a control. For the HepG2 *MDM4* overexpression experiment, the pCDH-CMV-MCS-EF1a-MDM4 lentiviral construct was generated by cloning the codon optimized cDNA sequence of MDM4 into the pCDH-CMV-MCS-EF1a vector (Epoch Life Science Inc., Missouri City, TX, USA). The empty pCDH-CMV-MCS-EF1a vector (pCDH-CMV-MCS-EF1a-vector control (vc)) was used as a control. All vector cloning was verified by sequencing with the following primers: 5-ggcgtgtacggtgggaggtct-3 (5′ sequencing primer), 5-tgtgggcgatgtgcgctctg-3 (3′ sequencing primer).

### Retroviral and lentiviral transduction of HB cell lines

For the *sh-MDM4* experiments, lentiviral supernatant was made by co-transfecting HEK-293 T cells with the packaging vectors hGPAM, Rev-1b, Tat-1b, and VSVG using XtremeGENE 9 DNA Transfection Reagent (Sigma-Aldrich). These viral particles were transduced into HepG2 and HepT1 cells with 8 μg/ml of hexadimethrine bromide (Polybrene, H9268, Sigma-Aldrich). Cells that showed at least 75% knock-down of *MDM4* gene expression with qPCR and immunoblotting experiments were then immediately used for experiments to examine effects on proliferation. Stable cell lines were not established with selection because strong knock-down of *MDM4* in the *sh-MDM4* cells resulted in strong proliferation defects and cell death that prevented stable cell lines from being established. For the *sh-TP53* experiments, lentiviral supernatant was made by co-transfecting HEK-293 T cells with the packaging vectors VSVG and pCMV using polyethylenimine (PEI) (1 μg/μl) added at a 3:1 ratio of PEI (μg):total DNA (μg). These viral particles were transduced into HepG2 and HepT1 cells with 8 μg/ml of hexadimethrine bromide (Polybrene, H9268, Sigma-Aldrich). Stable cell lines were established after one week of puromycin (2 μg/ml) selection. Knock-down was confirmed using immunoblotting. For the pBABE-MDM4 overexpression experiments, retroviral supernatant was made by co-transfecting HEK-293 T cells with the packaging vectors RDF and Pegpam3 using PEI (1 μg/μl) added at a 3:1 ratio of PEI (μg):total DNA (μg). These viral particles were transduced into HepT1 cells with 8 μg/ml of hexadimethrine bromide (Polybrene, H9268, Sigma-Aldrich). Stable cell lines were established after one week of puromycin (2 μg/ml) selection. Overexpression was confirmed using immunoblotting. For the pCDH-CMV-MCS-EF1a-MDM4 overexpression experiments, lentiviral supernatant was made by co-transfecting HEK-293 T cells with the packaging vectors VSVG and pCMV using polyethylenimine (PEI) (1 μg/μl) added at a 3:1 ratio of PEI (μg):total DNA (μg). These viral particles were transduced into HepG2 cells with 8 μg/ml of hexadimethrine bromide (Polybrene, H9268, Sigma-Aldrich). Stable cell lines were established after one week of puromycin (2 μg/ml) selection. Knock-down was confirmed using immunoblotting.

### Orthotopic mouse model

All animal procedures used in this study were performed under an animal protocol approved by the Institutional Animal Care and Use Committee (IACUC) of Baylor College of Medicine (AN-6191). All animal studies were also carried out in compliance with the ARRIVE guidelines. In vivo studies were performed in female NSG mice aged 6–8 weeks (Taconic Biosciences, Hudson, NY, USA). HepT1 cells transduced with *luciferase* were used such that intraperitoneal injection of luciferin into the animals resulted in the cells emitting a bioluminescence signal that could be monitored with the In Vivo Imaging System (IVIS, PerkinElmer, Waltham, MA, USA). For the HepT1 experiment, 2 × 10^6^ HepT1 cells transduced with *luciferase* and resuspended in 25 μl PBS mixed with 25 μl matrigel (354,230, Becton, Dickinson and Company) were surgically implanted into the right lobe of the liver of one animal through a right flank incision. Expression of strong luciferase activity (2–3 million RLU) was confirmed prior to implantation of the cells. After this mouse grew tumor, the animal was euthanized, and the tumor was cut into small pieces (approximately 3 mm^3^) and serially reimplanted into the livers of ten animals as has been done previously^28^. For this experiment, animals were added to treatment or placebo groups when they satisfied two criteria: (1) consecutive BLI scans with increasing flux values, indicating active tumor growth, and (2) flux value above a threshold of 7 × 10^6^ p/s, indicating tumor burden. Mice underwent BLI beginning at 10 days after implantation and twice every week thereafter with the IVIS, and luminescence flux was recorded to assess tumor growth. From 17 to 35 days, animals satisfied criteria, and were alternately added to NSC207895 (n = 5) and placebo (DMSO/saline) (n = 5) treatment groups. NSC207895 was given at a dose of 5 mg/kg every other day for three weeks. At the conclusion of treatment, all animals were euthanized and tissues were harvested for immunohistochemistry. All animals in this study were monitored on a daily basis for signs of pain and distress.

### In vivo* MRI*

MRI was performed on a 1.0 T permanent MRI scanner (M2 system, Aspect Technologies, Israel). A 35 mm volume coil was used for transmit and receive of radiofrequency (RF) signal. Mice were sedated using 3% isoflurane, setup on the MRI animal bed, and then maintained under anesthesia at 1–1.5% isoflurane delivered using a nose cone setup. Body temperature was maintained by circulating hot water through the MRI animal bed. Respiration rate was monitored using a pneumatically controlled pressure pad placed in the abdominal area underneath the animal. Tumor imaging for was performed using a T2-weighted fast-spin echo (FSE) sequence with the following scan parameters: echo time (TE) = 80 ms, repetition time (TR) = 3030 ms, slice thickness = 1.2 mm, field of view = 80 mm, number of slices = 16, matrix = 256 × 250, no. of signal averages = 2, dwell time = 25 μs, scan time ~ 2.5 min. Images were analyzed and processed in Osirix (version 5.8.5, 64-bit, Pixmeo, Bernex, Switzerland).

### Immunohistochemistry of intrahepatic tumor tissues

Tissue samples were fixed in 10% formalin (Alfa Aesar, Ward Hill, MA, USA) overnight at 4 °C. Tissues were then dehydrated in 70% ethanol until processing in paraffin. Samples were processed in the Texas Medical Center Digestive Diseases Center (Houston, TX, USA). Hematoxylin and eosin (H&E), TUNEL (S7100, Millipore), and Ki67 (CRM325C, Biocare Medical, Pacheco, CA, USA) stainings were performed. Imaging of tumor sections on slides was done on a BZ-X710 All-in-One Fluorescence Microscope (Keyence, Itasca, IL, USA). Quantification of TUNEL and Ki67 was done using the Hybrid Cell Count Analysis Application (Keyence).

### Statistical analysis

All values were presented as mean ± standard deviation (SD). Student’s t-Test (two-tailed) was used to determine statistical significance, and *p* values were indicated (*p* < 0.05 (*), *p* < 0.01 (**), *p* < 0.001 (***)). Kruskal Wallis test used to show significance of differences in tumor weights between treatment and placebo groups at time of euthanasia.

## Supplementary Information

Supplementary Information 1.
