# Author Correction: MDM4 inhibition: a novel therapeutic strategy to reactivate p53 in hepatoblastoma

**DOI:** 10.1038/s41598-021-98174-7

**Published:** 2021-10-01

**Authors:** Sarah E. Woodfield, Yan Shi, Roma H. Patel, Zhenghu Chen, Aayushi P. Shah, Rohit K. Srivastava, Richard S. Whitlock, Aryana M. Ibarra, Samuel R. Larson, Stephen F. Sarabia, Andrew Badachhape, Zbigniew Starosolski, Ketan B. Ghaghada, Pavel Sumazin, D. Allen Annis, Dolores López-Terrada, Sanjeev A. Vasudevan

**Affiliations:** 1 Divisions of Pediatric Surgery and Surgical Research, Michael E. DeBakey Department of Surgery, Pediatric Surgical Oncology Laboratory, Texas Children’s Surgical Oncology Program, Texas Children’s Liver Tumor Program, Dan L. Duncan Cancer Center, Baylor College of Medicine, Texas Children’s Hospital, 1102 Bates Ave., Suite 460G, Houston, TX 77030-2399 USA; 2 Department of Pathology and Immunology, Baylor College of Medicine, Molecular Oncology Laboratory, Texas Children’s Hospital, Houston, TX 77030 USA; 3 Singleton Department of Pediatric Radiology, Texas Children’s Hospital, Houston, TX 77030 USA; 4 Department of Pediatrics, Dan L. Duncan Cancer Center, Baylor College of Medicine, Houston, TX 77030 USA; 5 Aileron Therapeutics Inc, Cambridge, MA 02139 USA

Correction to: *Scientific Reports* 10.1038/s41598-021-82542-4, published online 03 February 2021

Rohit K. Srivastava was omitted from the author list in the original version of this Article.

The Author Contributions section now reads:

“Author contributions S.E.W., Y.S., R.H.P., S.F.S., and S.A.V. designed experiments. S.E.W., Y.S., R.H.P., Z.C., A.P.S., R.K.S., A.M.I., S.R.L., A.B., Z.S., P.S., and S.A.V. performed experiments. S.E.W., Y.S., R.H.P., R.S.W., P.S., and S.A.V. analyzed and interpreted data. S.E.W. and S.A.V. wrote the manuscript and figures. S.E.W., K.B.G., D.A.A., D.L.T., and S.A.V. supervised the study. S.A.V. conceived of the study and procured funding.”

The Acknowledgement section now reads:

“We would like to thank Dr. Igor Stupin for assistance with MRI. We would like to thank Pamela Parsons in the Texas Medical Center Digestive Diseases Center for completing the immunohistochemistry experiments. The Texas Medical Center Digestive Diseases Center is supported by NIH grant P30DK56338. We would like to thank Aileron Therapeutics for their generous gift of the ATSP-7041 compound used in this work. This work was supported by a Texas Children’s Department of Surgery Seed Award (S.A.V.), the Macy Easom Cancer Research Foundation Grant (S.A.V.), and a Cancer Prevention Research Institution of Texas (CPRIT) Multi-Investigator Research Award (RP180674, S.A.V.). This work was also supported in part by the European Union’s Horizon 2020 Research and Innovation Programme under grant agreement 826121 (P.S.) and by National Cancer Institute Award R21CA223140 (P.S.).”

The original Article has been corrected.

