# Supplementary material for: MDM4 inhibition: a novel therapeutic strategy to reactivate p53 in hepatoblastoma
Source: Sci Rep. 2021 Feb 3;11:2967. doi: 10.1038/s41598-021-82542-4 (PMC7859402; doi:10.1038/s41598-021-82542-4)
Supplement: Supplementary file 1 — Supplementary Information 1. [file 41598_2021_82542_MOESM1_ESM.pdf]

MDM4 Inhibition: A Novel Therapeutic Strategy to Reactivate p53 in Hepatoblastoma

Sarah E. Woodfield[1*], Yan Shi[1*], Roma H. Patel[1*], Zhenghu Chen[1], Aayushi P. Shah[1], Richard S. Whitlock[1], Aryana M. Ibarra[1], Samuel R. Larson[1], Stephen F. Sarabia[2], Andrew Badachhape[3], Zbigniew Starosolski[3], Ketan B. Ghaghada[3], Pavel Sumazin[4], D. Allen Annis[5], Dolores López-Terrada[2], and Sanjeev A. Vasudevan[1**]

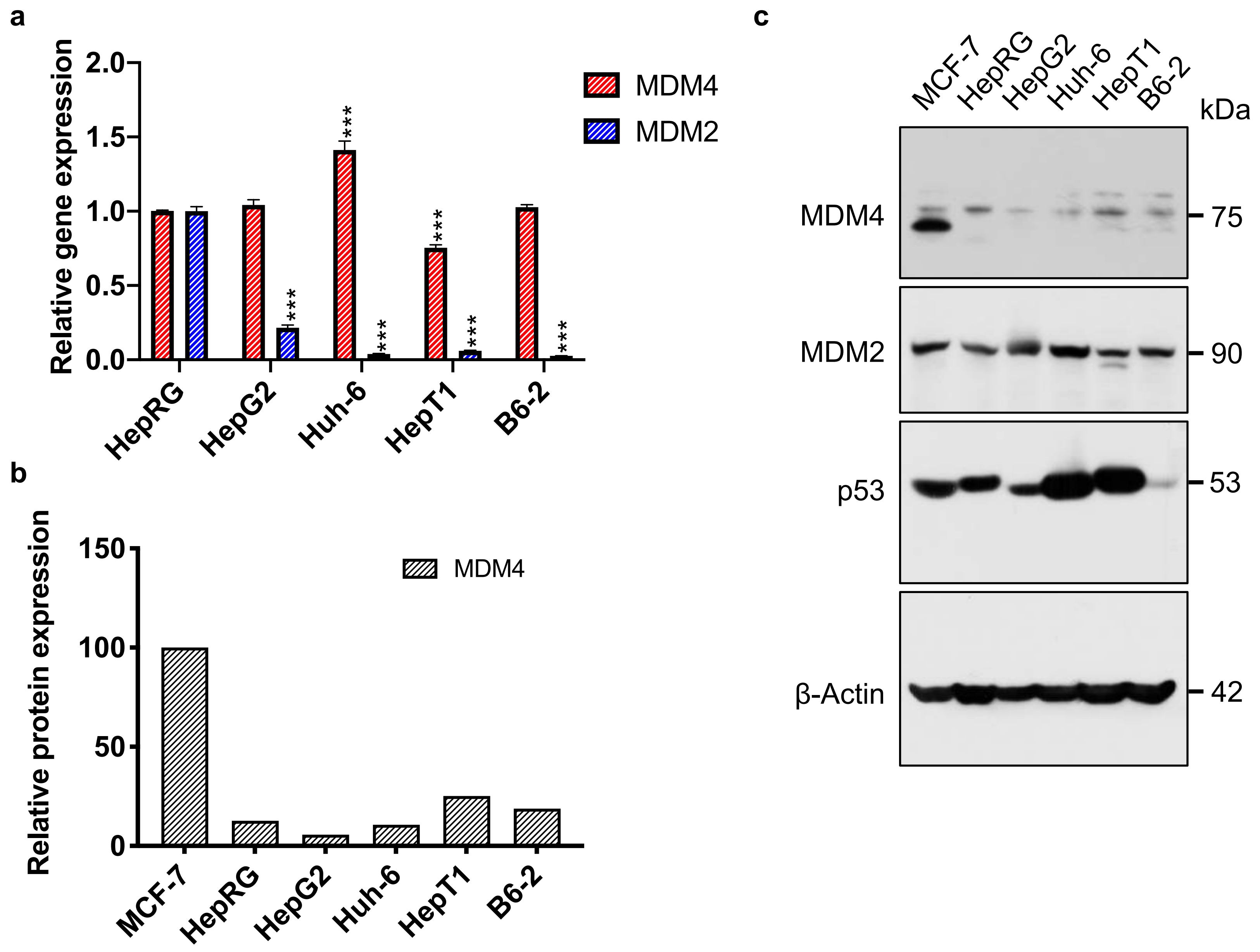

Supplementary Figure 1. HB cells show expression of MDM4. (a) Bar graphs representing normalized mRNA expression of *MDM4* and *MDM2* analyzed with qPCR experiments. Cell line expression data normalized to HepRG expression data. Error bars represent SD. Student's *t* test (two-tailed) **P<0.05*, ***P<0.01*, ****P<0.001*. Data shown are representative of at least three independent experiments performed with three replicate wells each time. **(b)** Densitometry analysis of the immunoblot shown in c. Protein expression data of cell lines normalized to protein expression data of MCF-7. **(c)** Immunoblotting with protein lysis from MCF-7, HepRG, and HB cells with antibodies recognizing MDM4 (NBP1-28862, Novus), MDM2, and p53. β-Actin immunoblotting was used as a loading control. Data shown are representative of at least three independent experiments.

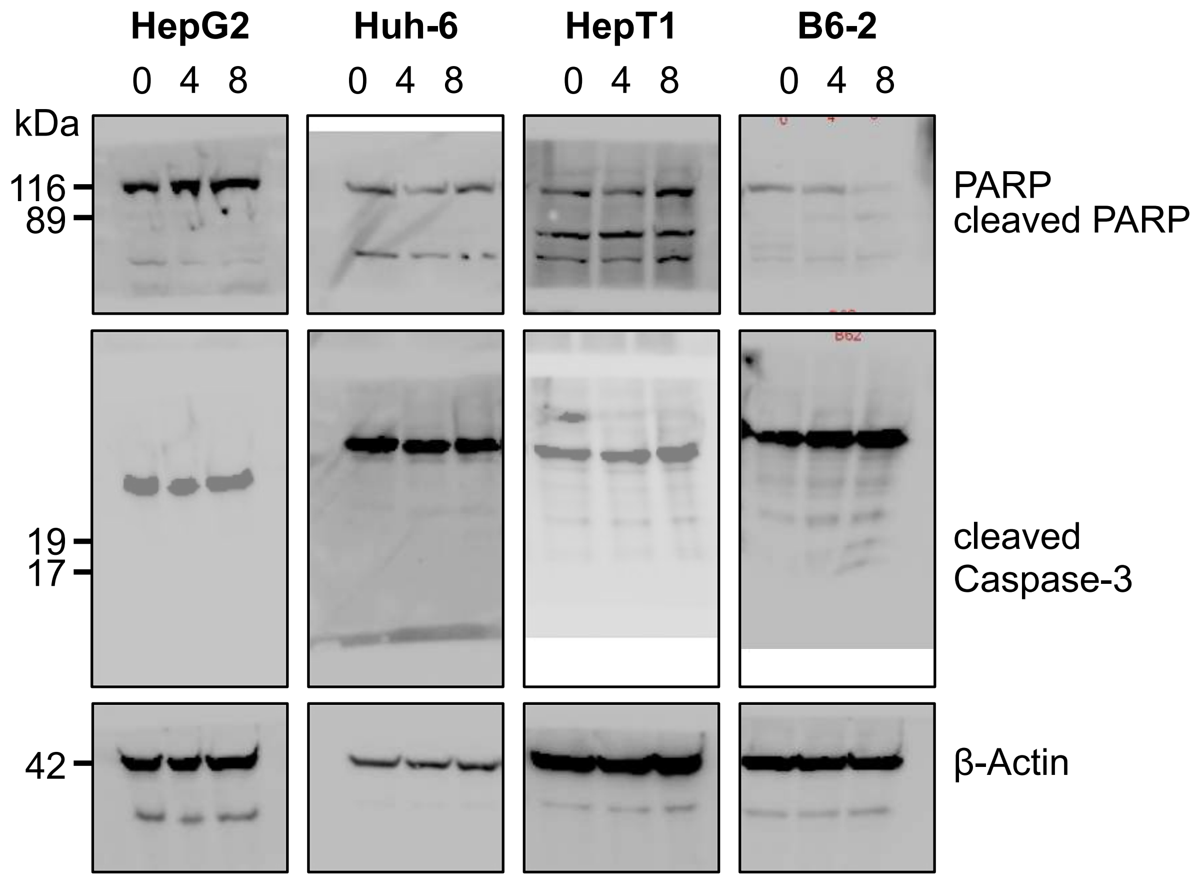

Supplementary Figure 2. PARP and Caspase-3 cleavage with Nutlin-3a treatment. Protein lysis from cells treated with 20 μM Nutlin-3a for 4 or 8 hours was compared to that from untreated cells (0 hours). Immunoblotting for total PARP, PARP cleavage, and Caspase-3 cleavage was done to assess apoptosis. β-Actin immunoblotting was used as a loading control. Data shown are representative of at least three independent experiments.

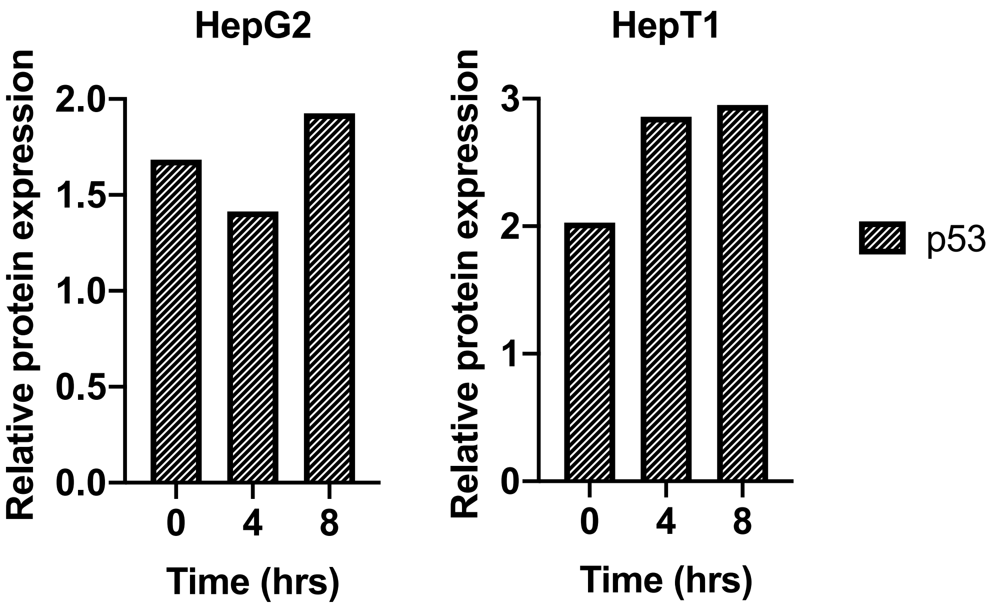

Supplementary Figure 3. MDM4 inhibition with NSC207895 alters p53 protein expression in HepT1 cells but not in HepG2 cells. Densitometry analysis of the immunoblot shown in Figure 3d.

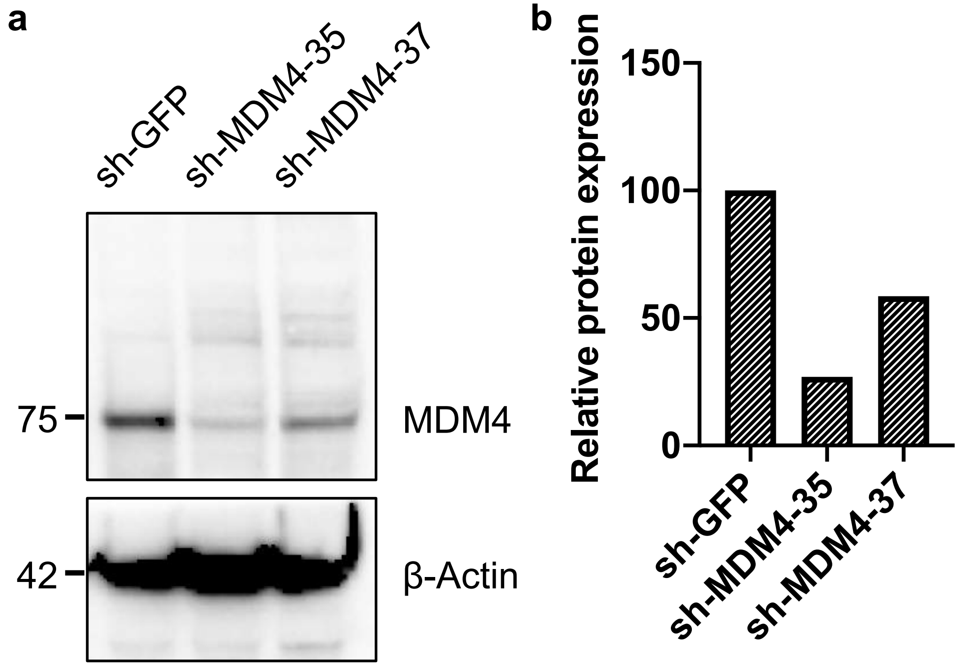

Supplementary Figure 4. MDM4 downregulation in transduced HepG2 cells. (a) Immunoblotting with HepG2 cells transduced with *sh-GFP*, *sh-MDM4-35*, or *sh-MDM4-37* with an antibody recognizing MDM4 (04-1555, Millipore). β-Actin immunoblotting was used as a loading control. Data shown are representative of at least three independent experiments. **(b)** Densitometry analysis of the immunoblot shown in a. Protein expression data of cell lines normalized to protein expression data of *sh-GFP*.

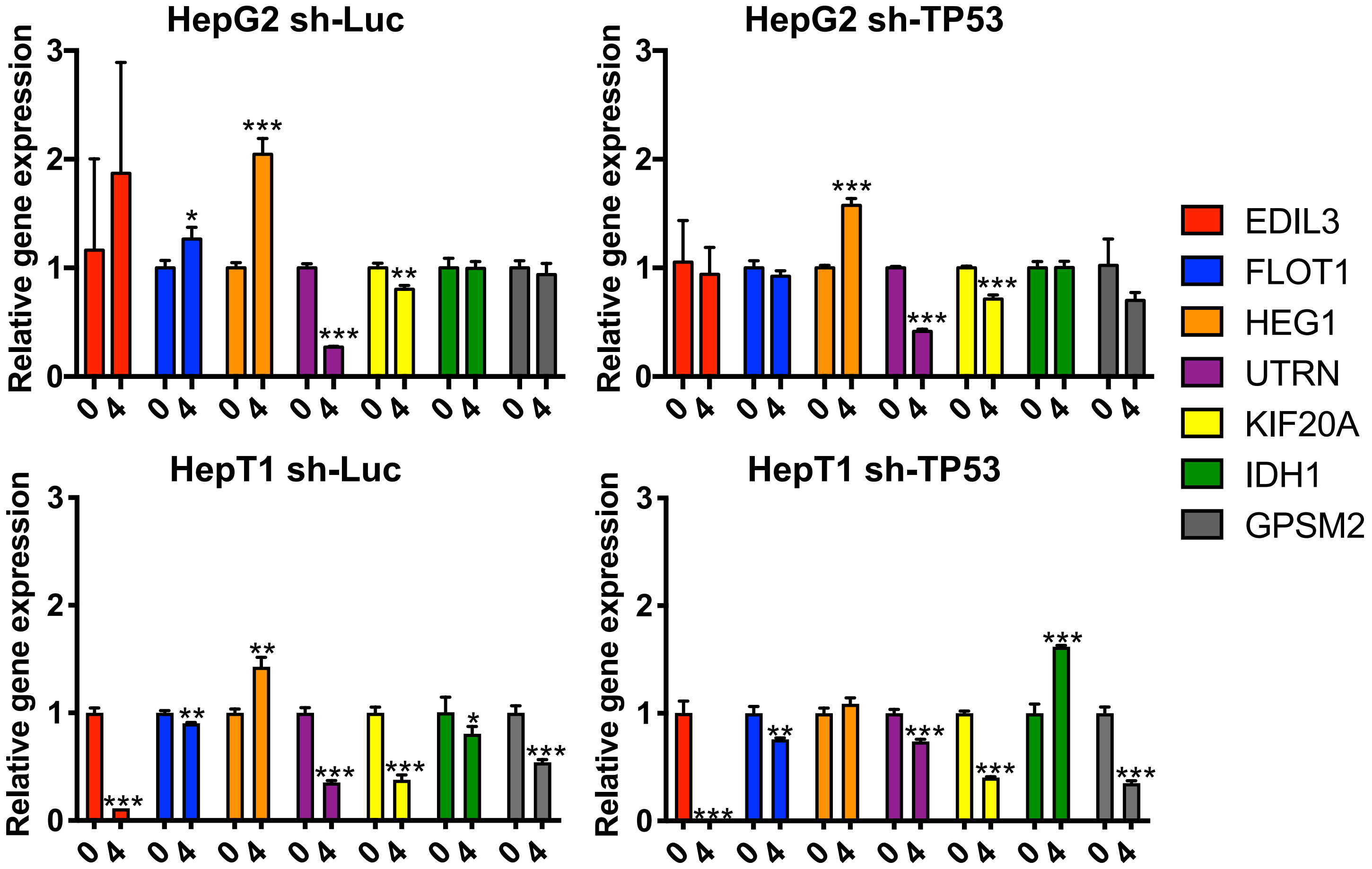

Supplementary Figure 5. Proposed alternative targets of NSC207895 are inconsistently affected or affected in a *TP53* dependent manner in HepG2 and HepT1 cells. Bar graphs represent normalized mRNA expression of *EDIL3*, *FLOT1*, *HEG1*, *UTRN*, *KIF20A*, *IDH1*, and *GPSM2* analyzed with qPCR experiments. RNA was extracted from HepG2 and HepT1 cells transduced with *sh-TP53* or control *sh-Luc* exposed to 10 μM NSC207895 for 4 hours, and this expression was compared to RNA from untreated (0 hours) cells. Data shown are representative of at least three independent experiments performed with three replicate wells each time. Error bars represent SD. Paired *t* test (two-tailed) *$P<0.05$, **$P<0.01$, ***$P<0.001$.

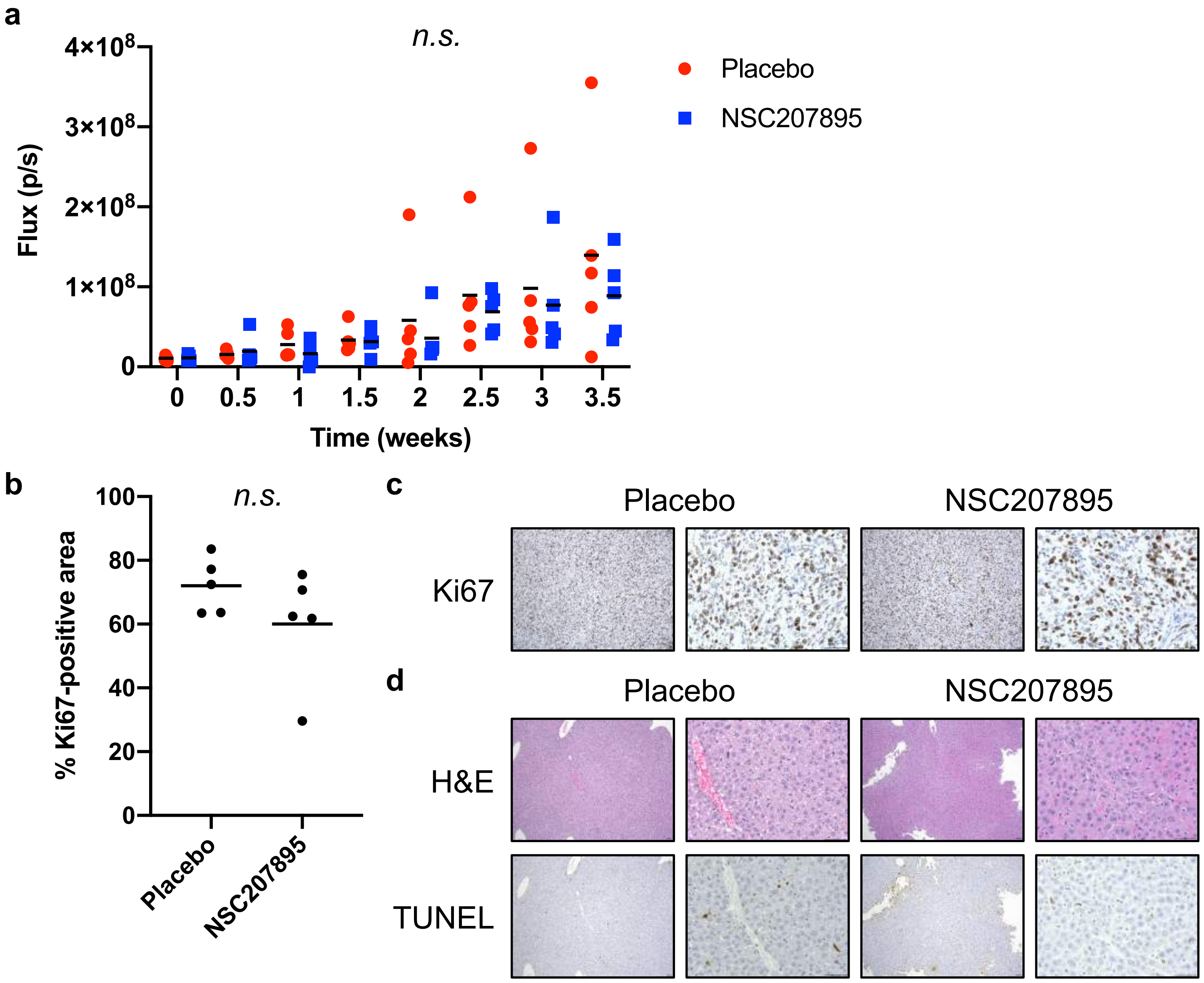

Supplementary Figure 6. *In vivo* study of inhibition of MDM4 with NSC207895. (a) BLI data showing growth of tumors with NSC207895 or placebo treatment over the course of the study. **(b)** Quantification of Ki67 staining of the tumors from the placebo and treatment animals. Ki67-positive areas from images taken on a Keyence BZ-X710 All-in-One Fluorescence Microscope were quantified using the Keyence Hybrid Cell Count Analysis Application. Bars indicate mean. **(c)** Representative pictures of Ki67 staining quantified in b. **(d)** Representative pictures of H&E and TUNEL staining. Scale bars represent 50 μM in 10X and 40X images in c and d.

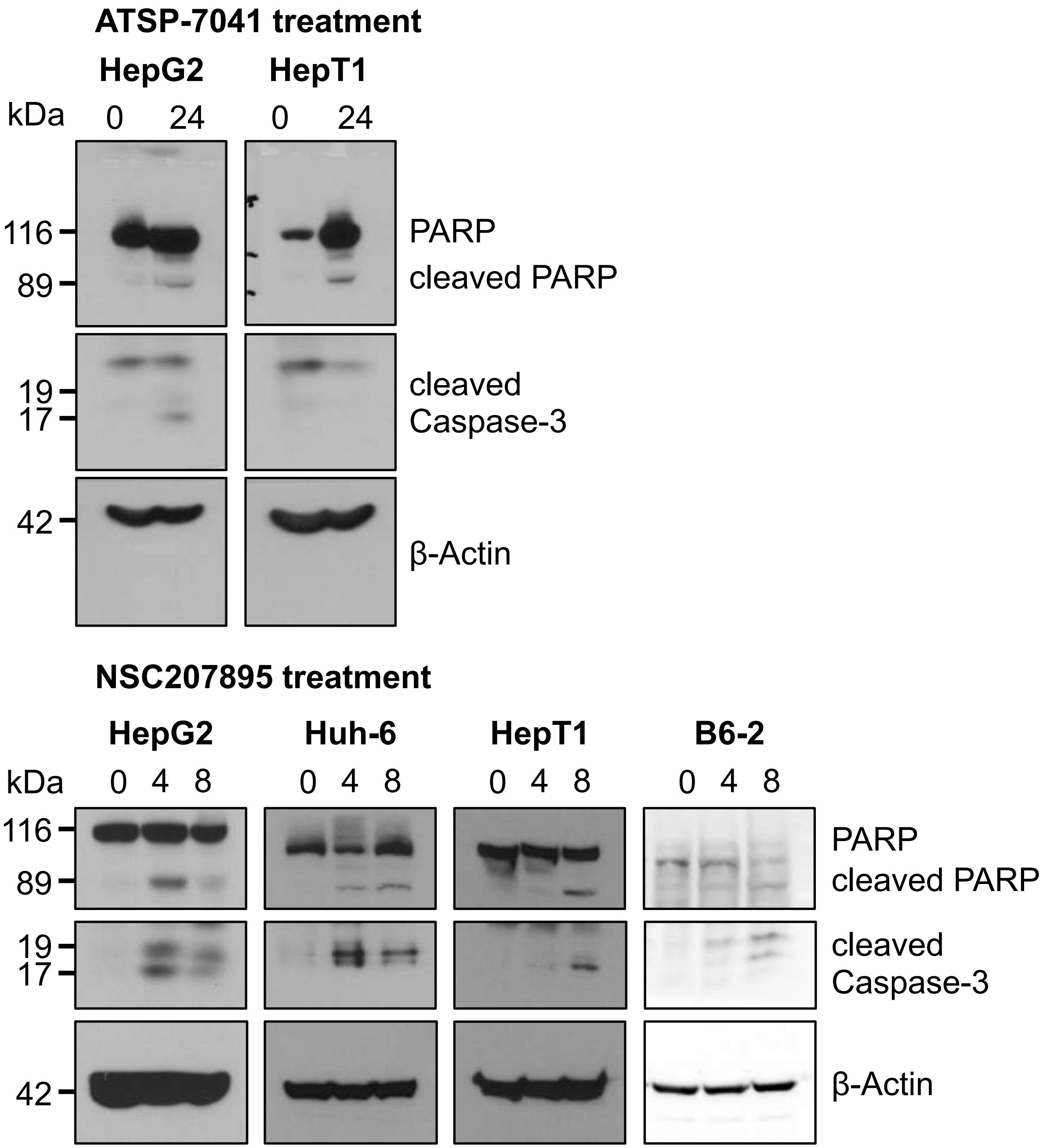

Supplementary Figure 7. Extended immunoblotting images from Figure 2e, f.

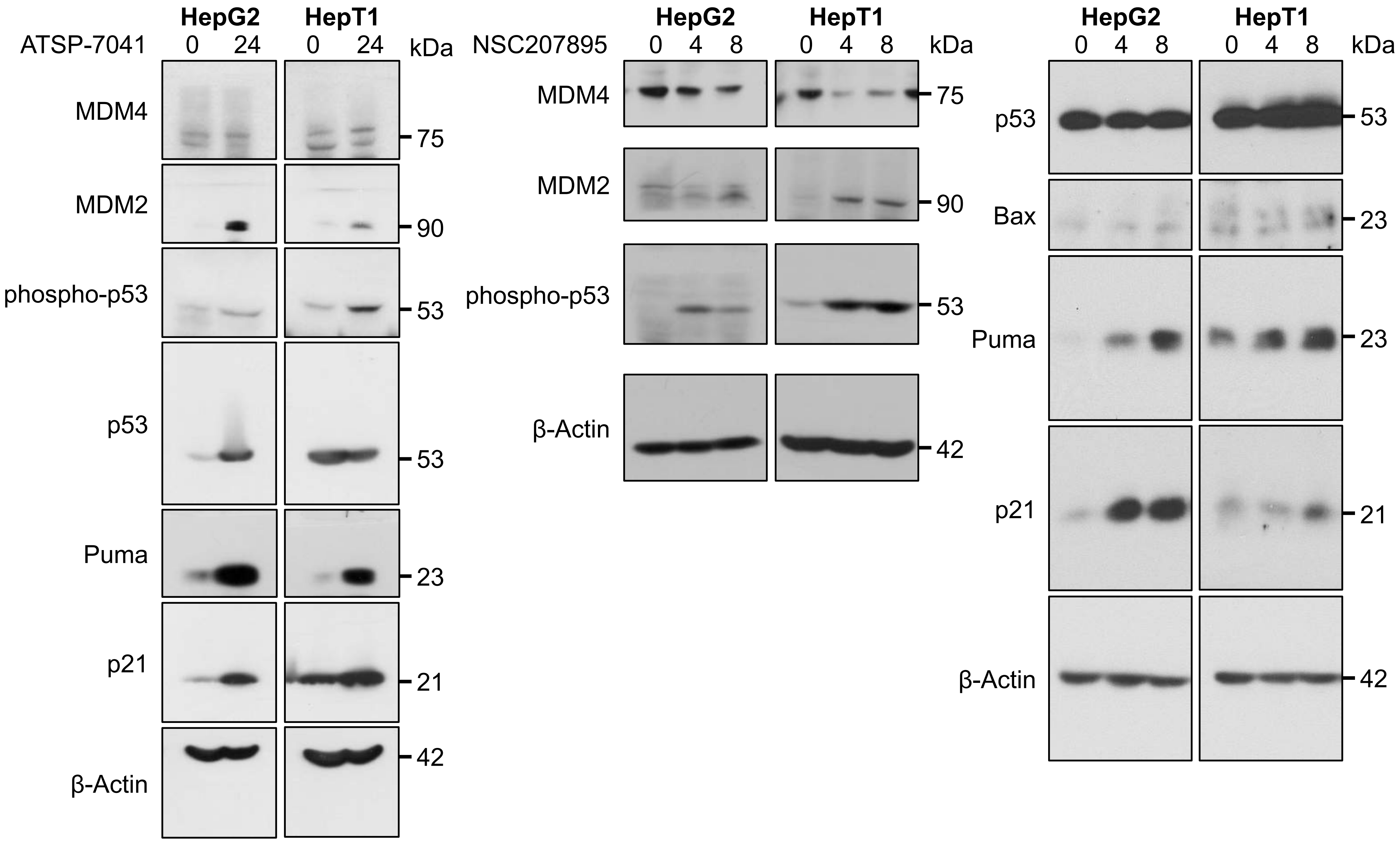

Supplementary Figure 8. Extended immunoblotting images from Figure 3c,d.

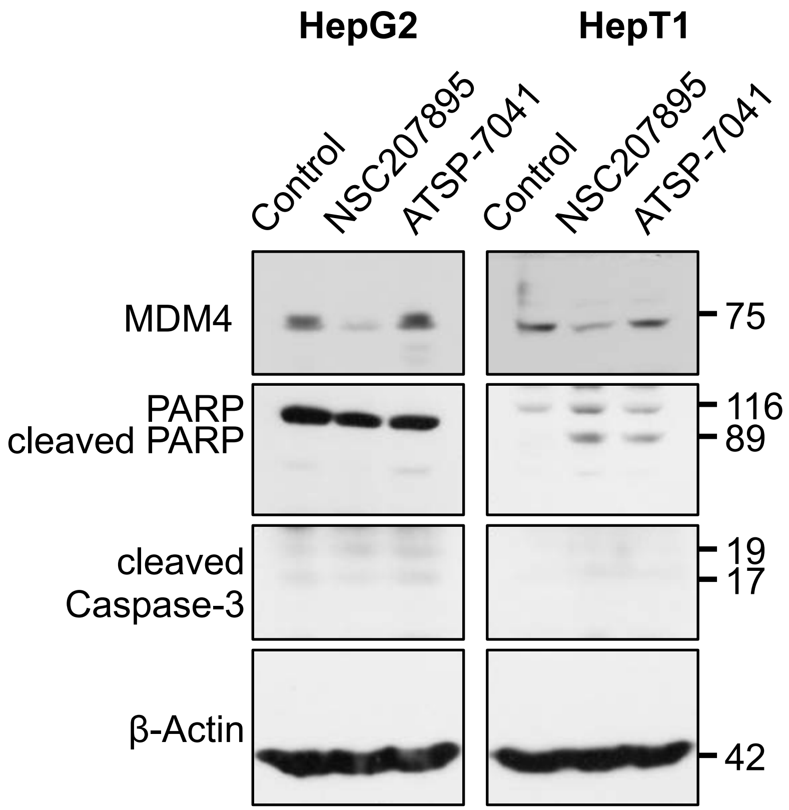

Supplementary Figure 9. Extended immunoblotting images from Figure 5c.

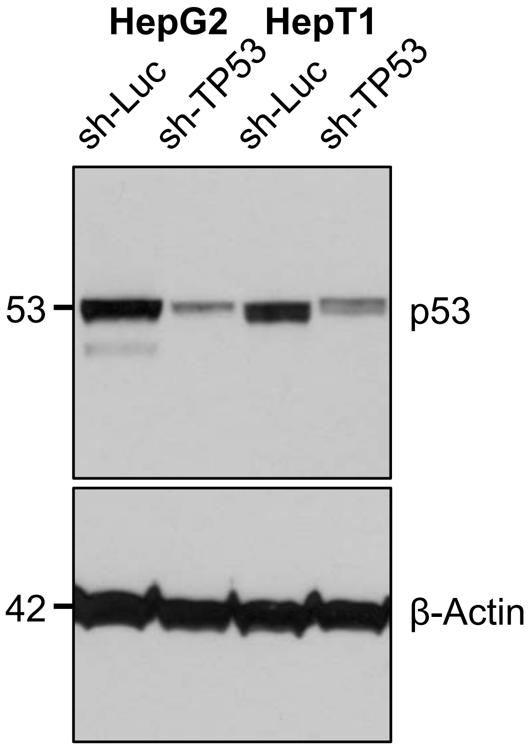

Supplementary Figure 10. Extended immunoblotting images from Figure 6a.

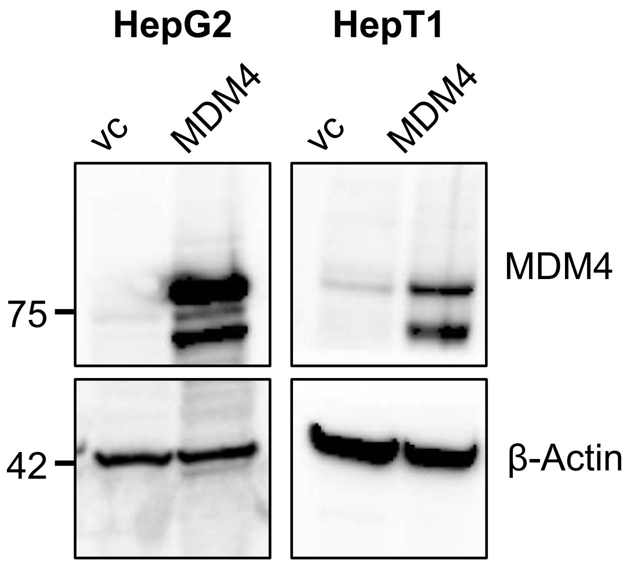

Supplementary Figure 11. Extended immunoblotting images from Figure 7a.

	HepG2	HepT1
0.05	100	>91.32
0.1	82.34	91.32
0.3	>72.19	>78.44
2	37.3	>63.47

Supplementary Table 1. Estimated percent viabilities of cells exposed to sub-IC50 concentrations (μM) of ATSP-7041 for 48 hours.

	HepG2	HepT1
0.05	>60.49	>85.55
0.1	60.49	85.55
0.3	>52.89	>76.65

Supplementary Table 2. Estimated percent viabilities of cells exposed to sub-IC50 concentrations (μM) of NSC207895 for 48 hours.

	IC_{50} (μM): sh-Luc	IC_{50} (μM): sh-TP53	IC_{50} (μM): vc	IC_{50} (μM): MDM4
HepG2	4.01	9.94	5.3	>10
HepT1	1.75	>10	1.19	3.16

Supplementary Table 3. Shifts in IC50 values with downregulation of TP53 and upregulation of MDM4. IC50 values determined in the assays shown in Figures 5b and 6c.
